# Supplementary material for: Stem rust resistance in wheat is suppressed by a subunit of the mediator complex
Source: Nat Commun. 2020 Feb 28;11:1123. doi: 10.1038/s41467-020-14937-2 (PMC7048732; doi:10.1038/s41467-020-14937-2)
Supplement: Supplementary file 1 — Supplementary Information [file 41467_2020_14937_MOESM1_ESM.pdf]

Stem rust resistance in wheat is suppressed by a subunit of the Mediator complex

Hiebert et al.

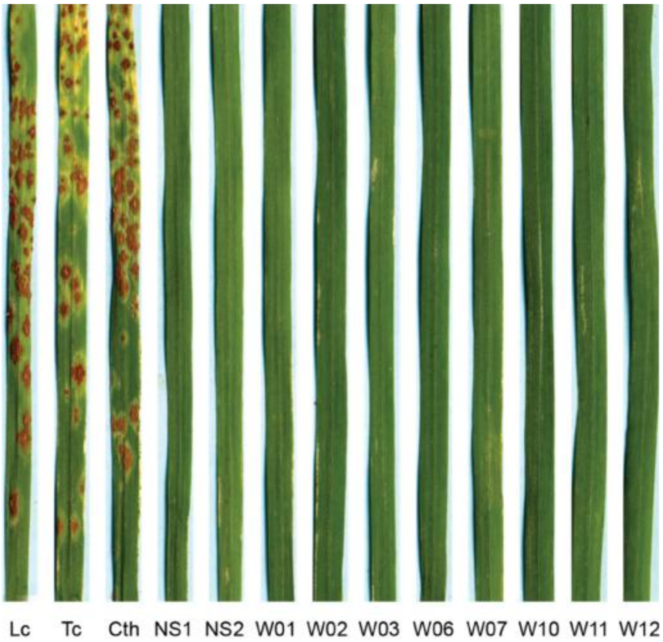

Supplementary Figure 1 | Extended panel of mutants in *SuSr-D1* with activated wheat stem rust resistance. Infection types of first leaves of seedlings inoculated with *Pgt* race QTHJC 14 days post-inoculation using an extended Canthatch (CTH) mutant panel[26]. Little Club (Lc) is the susceptible control. NS1, NS2, and mutants identified by Williams *et al*. (1992) (prefix W) showed no visible symptoms, whereas CTH and Thatcher (Tc) showed susceptible responses characterized by large uredinia (pustules).

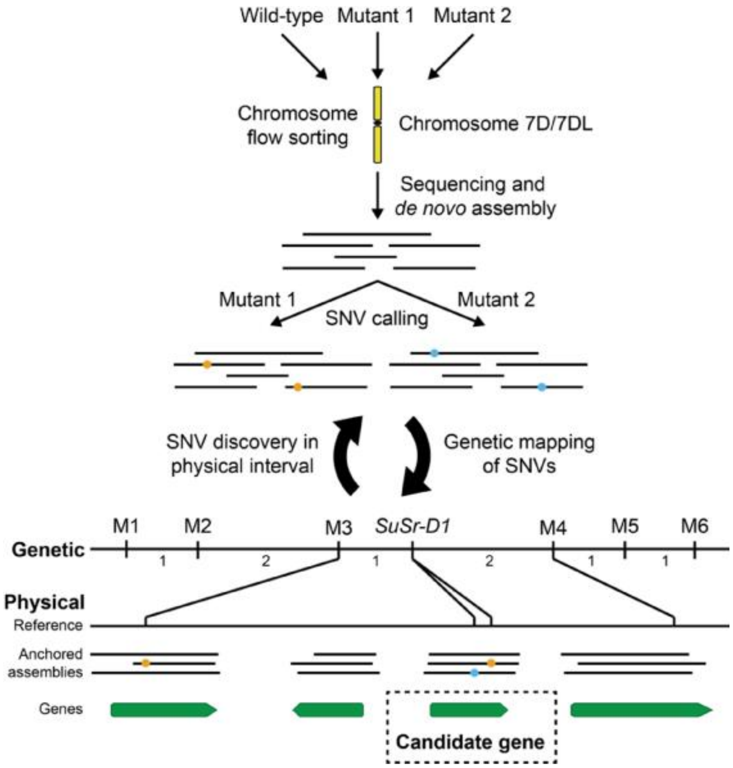

Supplementary Figure 2 | Integration of SNV discovery and genetic mapping to isolate *SuSr-D1*. A generalized pipeline to identify genes of interest in wheat. Chromosomes or chromosome arms are isolated using flow sorting, sequenced, and used to generate *de novo* assemblies. SNV calling using the wild-type reference forms the basis for marker development. SNV discovery and iterative genetic mapping are used to narrow down the gene of interest, here *SuSr-D1*.

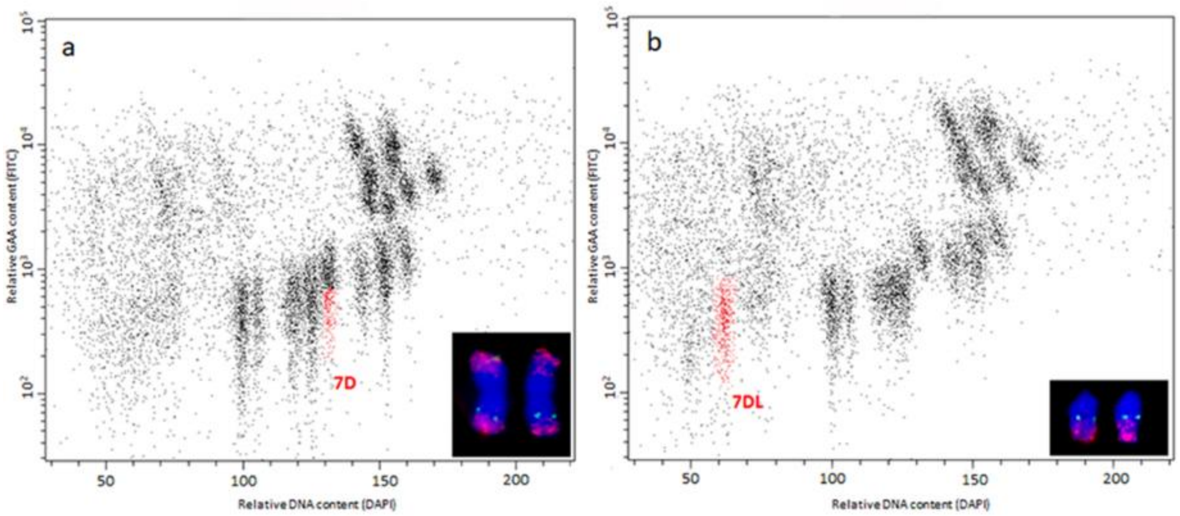

Supplementary Figure 3 | Bivariate flow karyotypes obtained after flow cytometric analysis of chromosomes isolated from (a) NS2 and (b) CTH-DT7DL lines. Prior to the analysis, GAA microsatellites were labelled by FITC using FISHIS, and chromosomal DNA was stained by DAPI. Sorted regions are highlighted in red. Inset: Images of flow-sorted chromosomes 7D (a) and chromosome arms 7DL (b) after FISH with probes for GAA microsatellites (green) and Afa-family repeat (red). Fluorescent labelling patterns served to identify sorted chromosomes and estimate the extent of contamination of sorted fractions by other chromosomes. Chromosomes were counterstained by DAPI (blue).

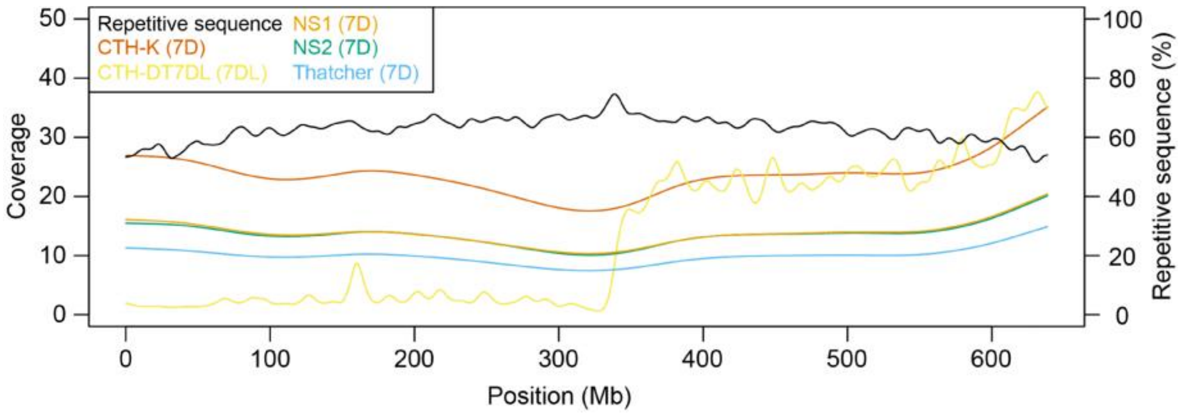

Supplementary Figure 4 | Coverage and repetitive sequence composition of wheat chromosome 7D. Genomic reads from flow sorted chromosomes or chromosome arms from CTH-K, CTH-DT7DL, NS1, NS2, and Thatcher were aligned to repeat masked IWGSC v1.0 chromosome 7D. A window-based analysis of coverage was applied to identify average coverage over the chromosome (coloured lines, left axis). Repetitive sequence is shown in black (right axis).

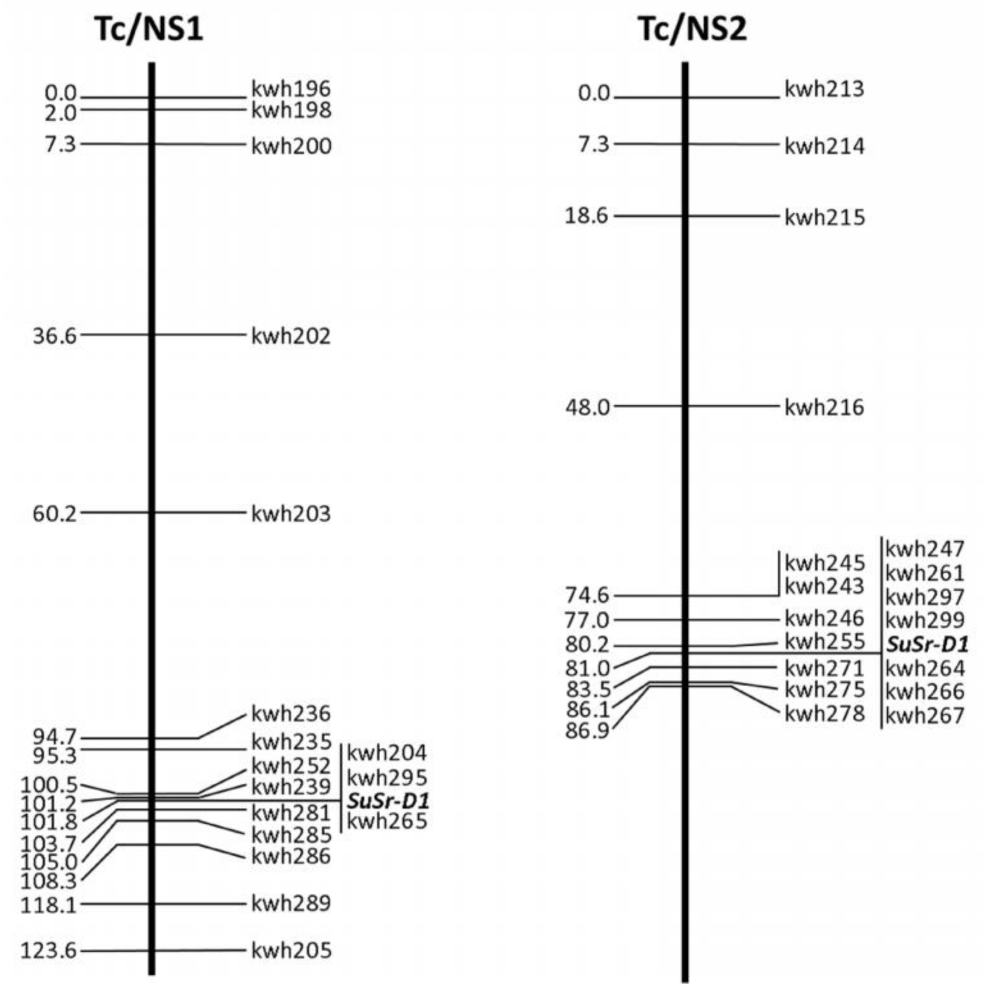

Supplementary Figure 5 | Linkage maps for the Thatcher (Tc) x NS1 and Tc x NS2 DH populations showing the position of *SuSr-D1* relative to markers based on EMS-induced SNVs. Genetic distances are in cM.

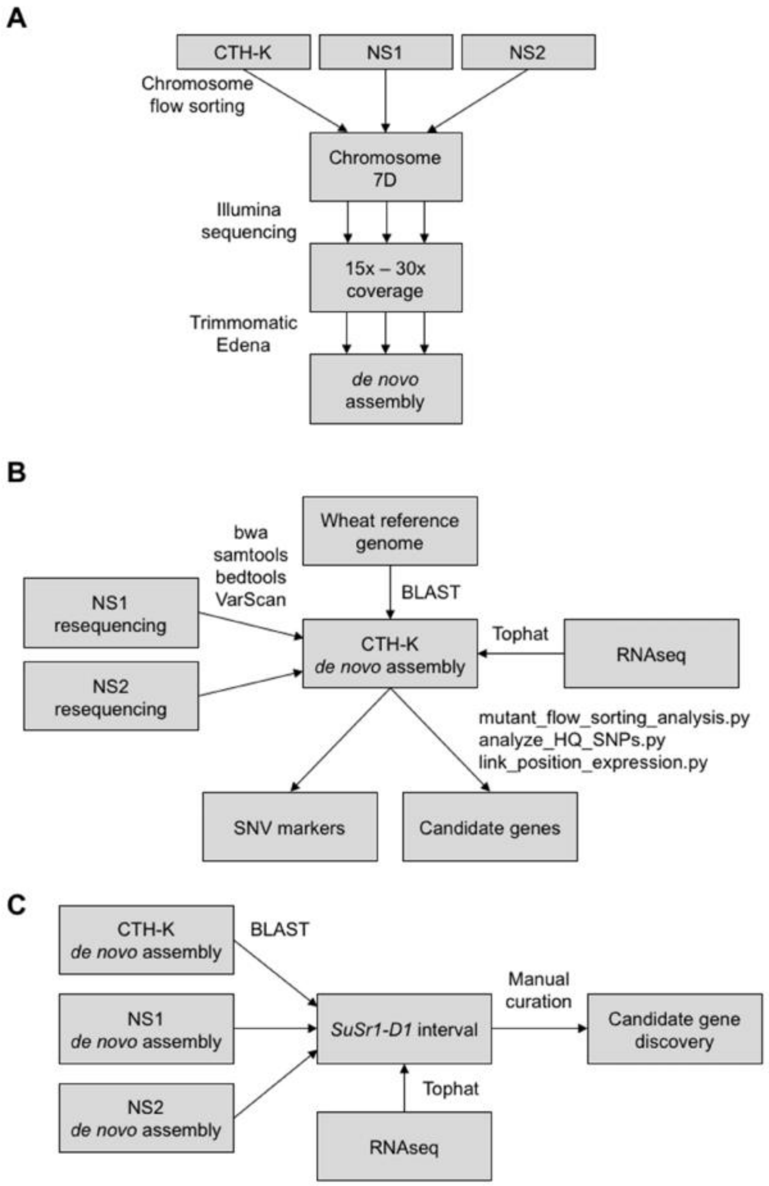

Supplementary Figure 6 | Flow chart of sequencing and bioinformatics approach used to identify SNV markers for linkage mapping and candidate genes. (**A**) Flow sorting chromosome 7D and/or chromosome arm 7DL was used for genome complexity reduction prior to Illumina sequencing. Independent *de novo* assemblies were made for the NS1 and NS2 mutant lines and wildtype CTH-K. (**B**) Candidate genes were identified through the integration of EMS-derived SNVs in NS1 and NS2, RNAseq-based gene discovery, and anchoring to the wheat reference genome. Intregration of these multiple data sources was achieved through custom Python scripts available at Github (https://github.com/matthewmoscou/Canthatch). (**C**) Manual curation of the *SuSr-D1* interval was achieved through alignment of independent CTH-K, NS1, and NS2 *de novo* assemblies. RNAseq was used for gene annotation in parallel with existing annotations from the wheat reference genome.

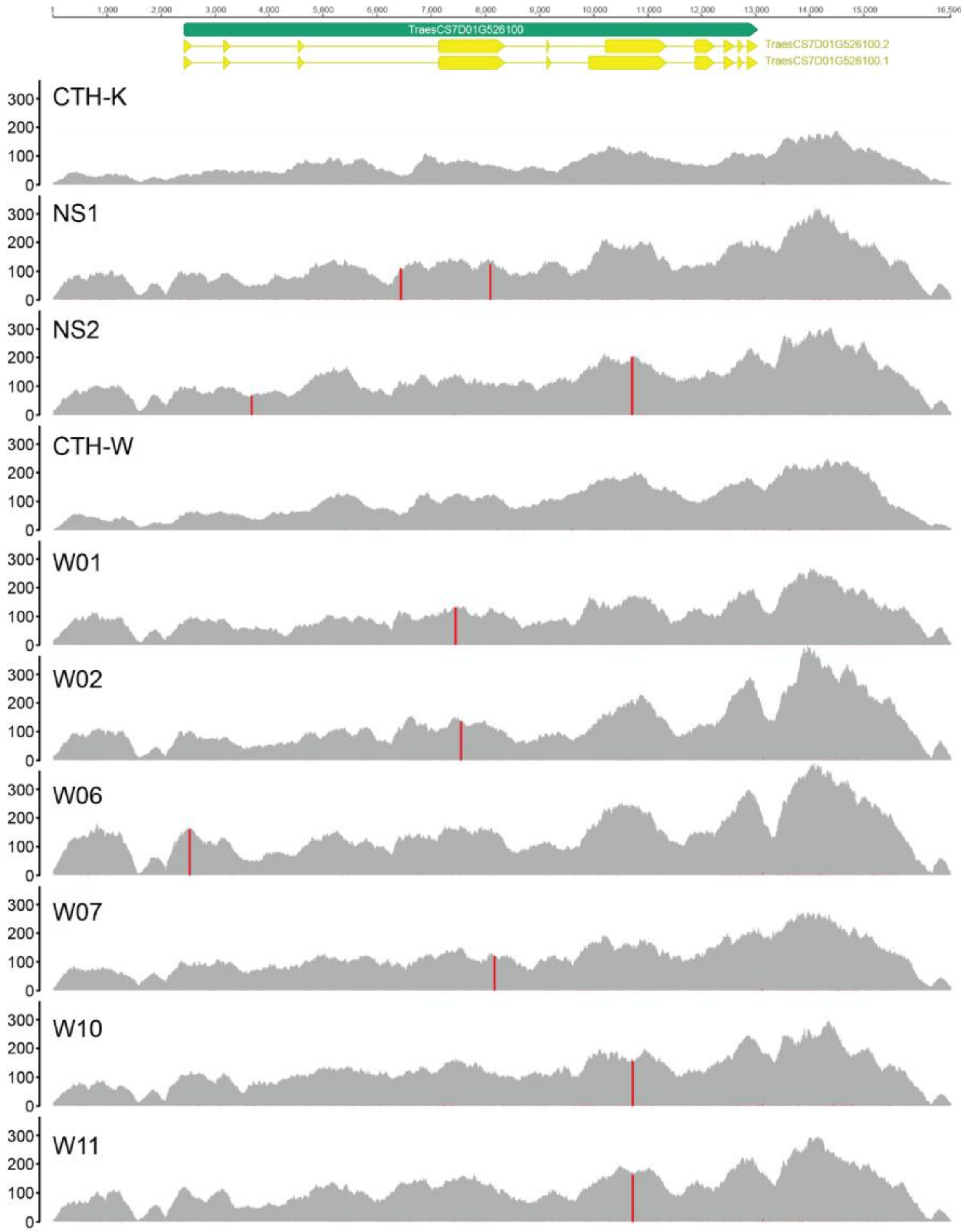

Supplementary Figure 7 | Alignment of reads from flow-sorted chromosome 7D to the contig containing *Med15b.D*. Top panel shows the gene model of *Med15b.D*. Bottom panels show variants for each sequenced mutant relative to the CTH reference in red, x-axis and y-axis are individual positions in the contig and read coverage, respectively.

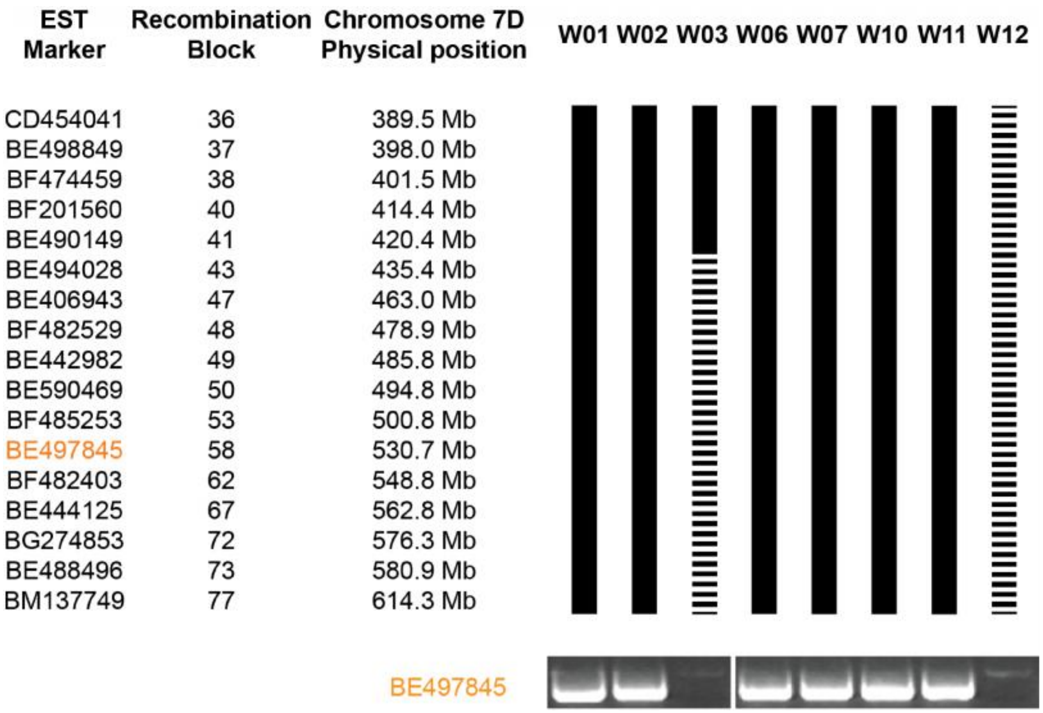

Supplementary Figure 8 | Identification of CTH mutants with large deletions in the Williams *et al*. series[26]. Markers from expressed sequenced tags (ESTs) mapping to chromosome arm 7DL with recombination block and physical position on IWGSC RefSeq v1.0[92]. W12 mutant was previously reported as ditelosomic 7DL [26]. Non-amplifying regions are shown as dashed chromosome segments. An example of marker BE497845 is shown in the bottom panel with a split gel image from PCR. The primer sequences were derived from Akhunov et al. (2010)[92].

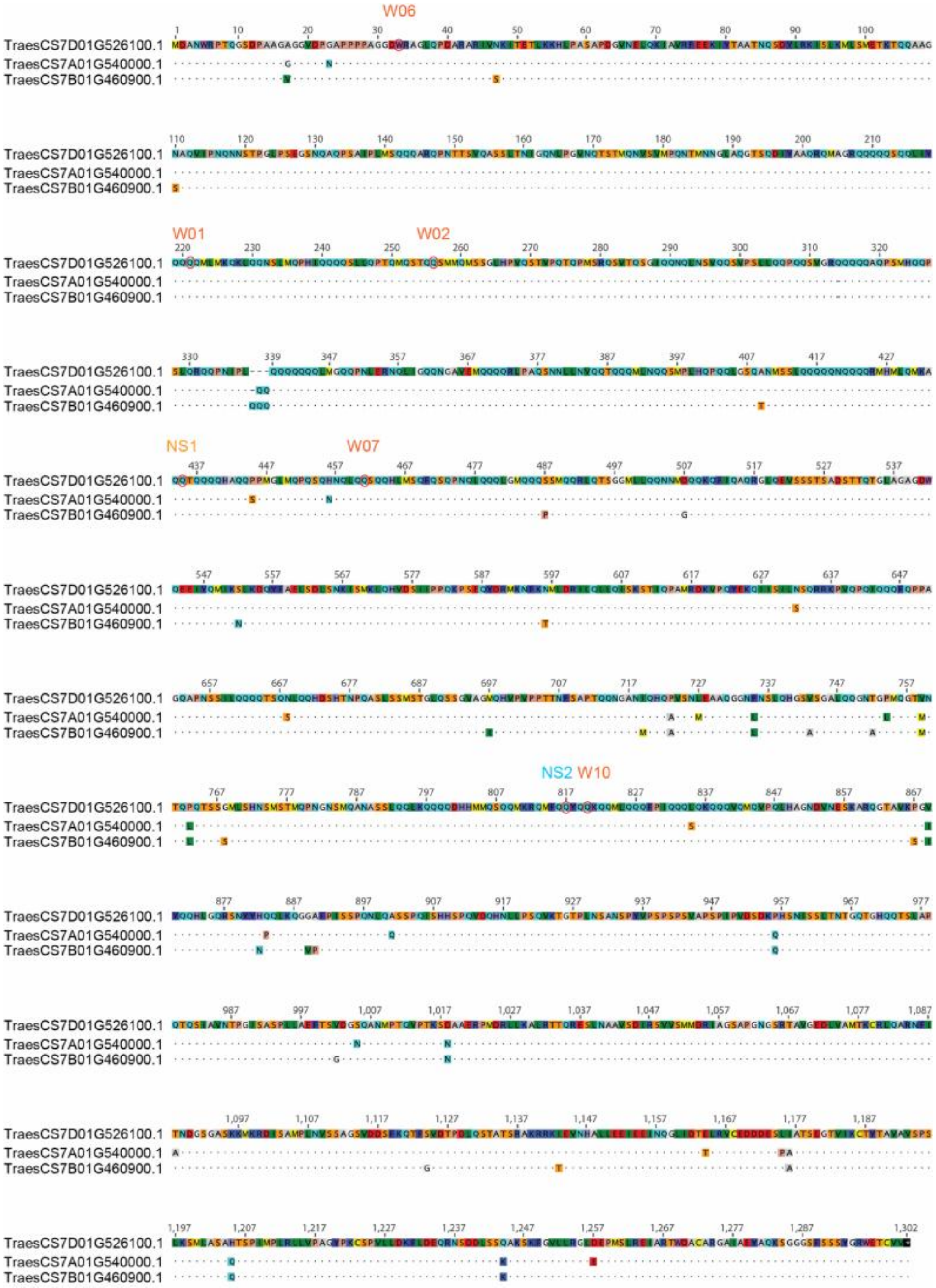

Supplementary Figure 9 | Protein alignment of *Med15b* homeologs on long arms of homeologous group 7 chromosomes. Sites of nonsense mutations are denoted based on the mutant line.

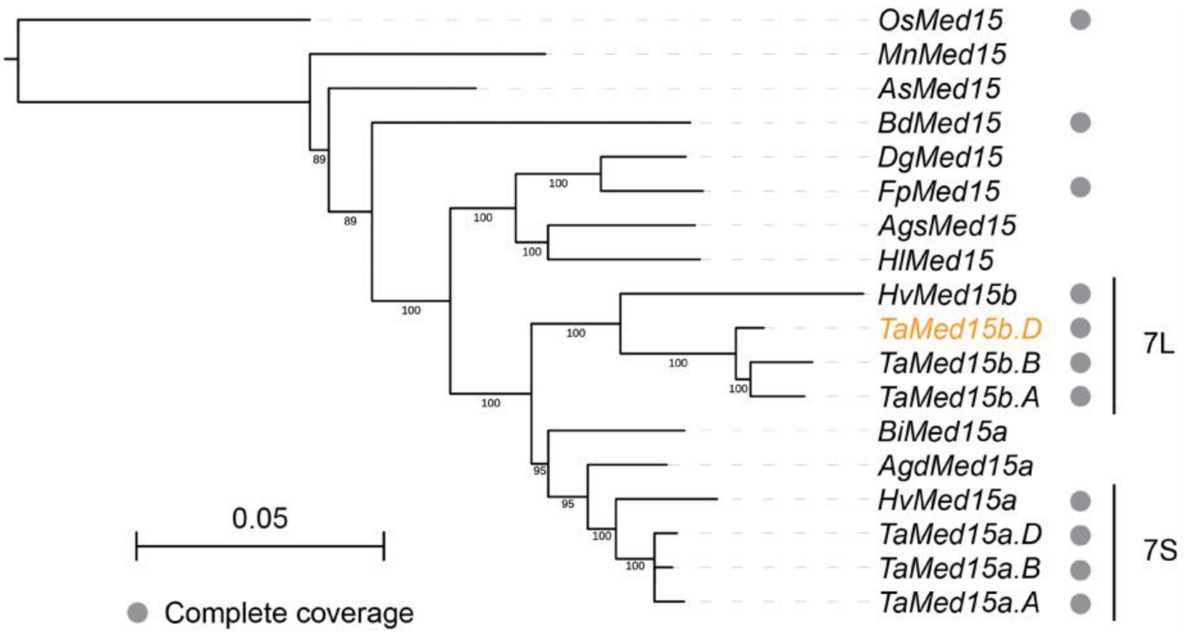

Supplementary Figure 10 | Maximum likelihood phylogenetic tree of *Med15* homologs in the grasses. PRANK was used for codon-based DNA alignment and the tree was constructed using RAxML using the GTRCAT model with 2,000 bootstraps. Full coding sequences were available for species denoted by grey circles. Unit of distance is nucleotide substitutions per evaluated sites. *SuSr-D1* (*TaMed15b.D*) is in orange script. *OsMed15* was used as an outgroup. Species include *Achnatherum splendens* (*As*), *Agropyron desertorum* (*Agd*), *Agrostis stolonifera* (*Ags*), *Brachypodium distachyon* (*Bd*), *Bromus inermis* (*Bi*), *Dactylis glomerata* (*Dg*), *Festuca pratensis* (*Fp*), *Holcus lanatus* (*Hl*), barley (*Hordeum vulgare; Hv*), *Melica nutans* (*Mn*), rice (*Oryza sativa; Os*), wheat (*Triticum aestivum*; *Ta*).

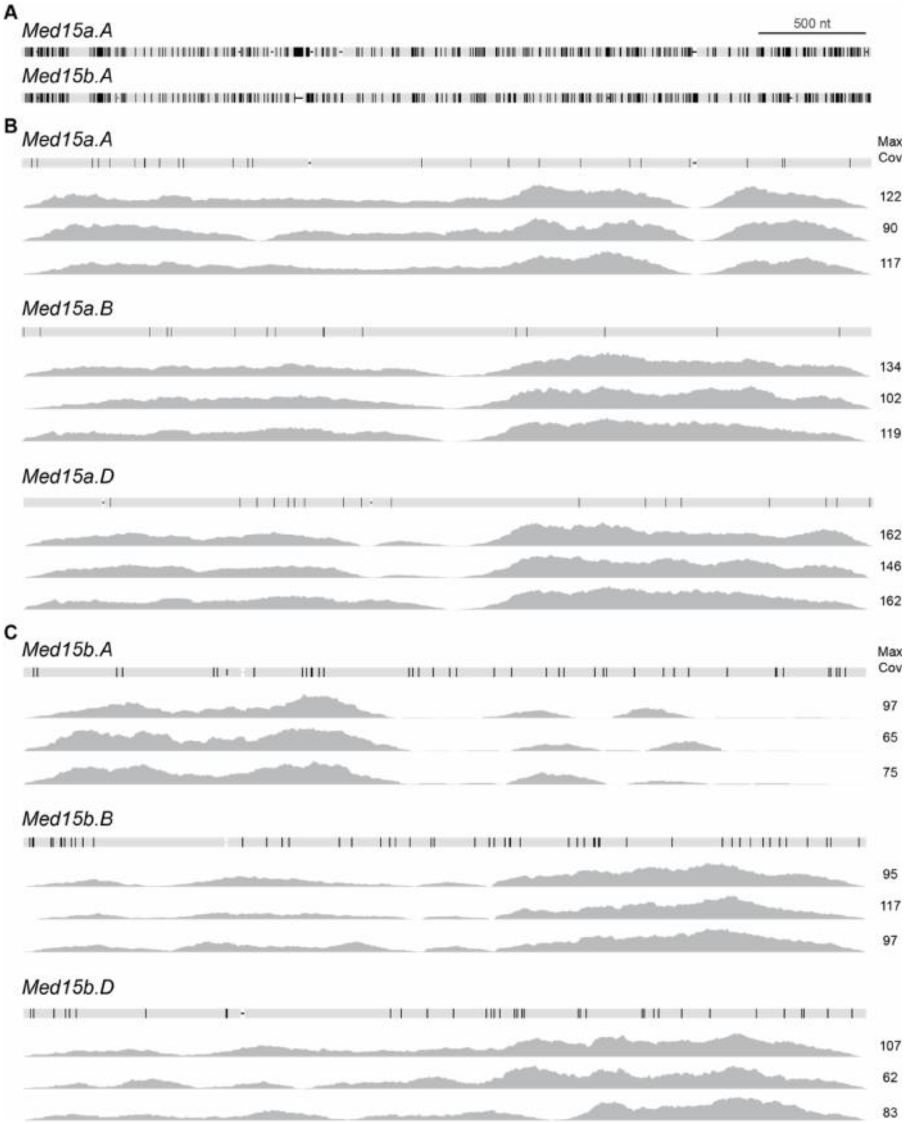

Supplementary Figure 11 | All members of the *Med15* gene family are expressed in leaves. (a) Alignment of the open reading frames of *Med15a.A* and *Med15b.A*. (b) and (c) Depth of coverage of aligned RNAseq data using maximum sensitivity (BBmap, 100% identity, unambiguous mapping, proper paired reads only) to the open reading frames of the *Med15a* and *Med15b* gene families, respectively. In all panels, identical and variant nucleotides are shown in grey boxes and black lines, respectively, insertions/deletions are shown as dashes, and maximum coverage (max cov) of a nucleotide position in each RNAseq analysis is shown on the right.

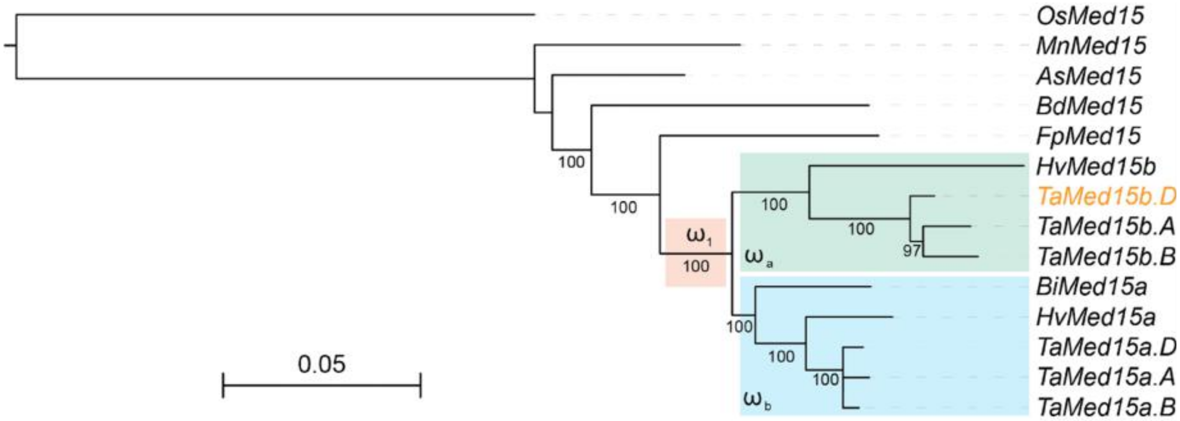

Supplementary Figure 12 | *Med15* maximum likelihood phylogenetic tree used for molecular evolutionary analyses. PRANK was used for codon-based DNA alignment and the tree was constructed using RAxML and the GTRCAT model with 2,000 bootstraps. A requirement of 90% coverage was needed for molecular evolutionary analyses. Branch and clade-based estimation of ω (d_N/d_S) are highlighted (Extended Data Table 2). Unit of distance is nucleotide substitutions per evaluated sites. *SuSr-D1* (*TaMed15b.D*) is denoted in orange script. *OsMed15* was used as an outgroup. Species include *Achnatherum splendens* (*As*), *Brachypodium distachyon* (*Bd*), *Bromus inermis* (*Bi*), *Festuca pratensis* (*Fp*), barley (*Hordeum vulgare*; *Hv*), *Melica nutans* (*Mn*), rice (*Oryza sativa*; (*Os*), wheat (*Triticum aestivum*; *Ta*).

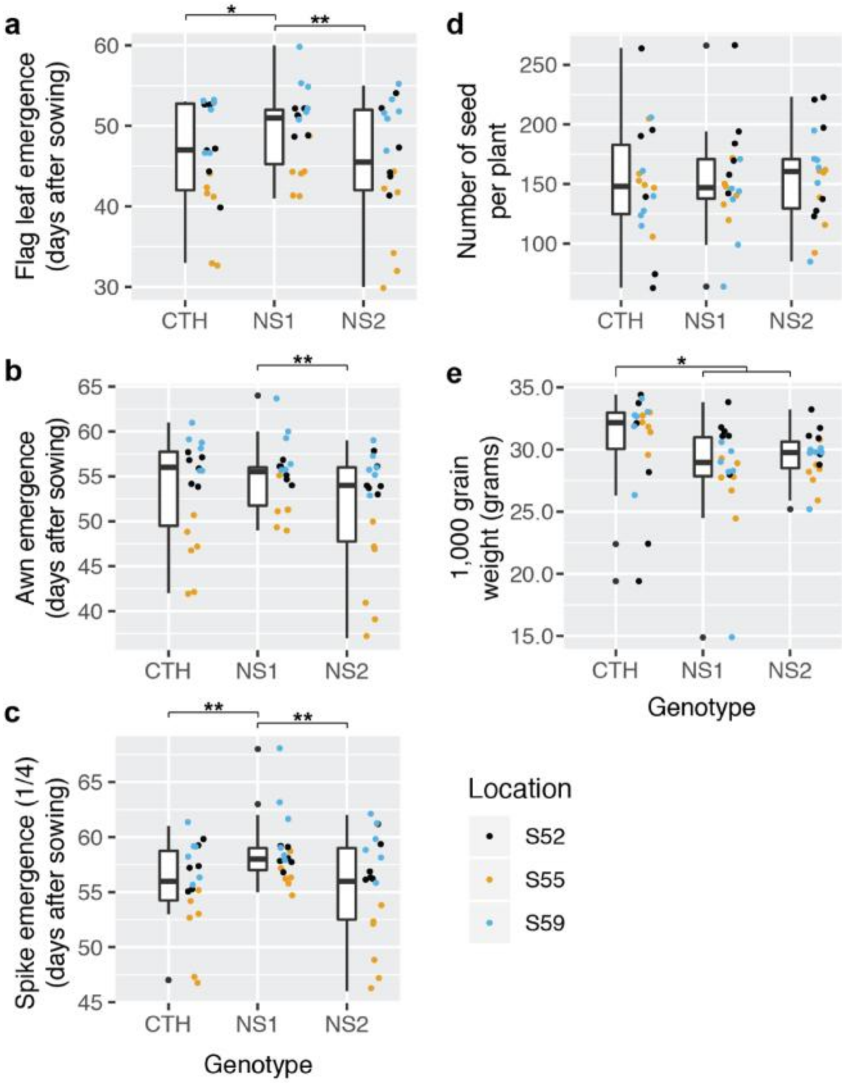

Supplementary Figure 13 | Assessment of plant developmental parameters of CTH, NS1, and NS2. Six plants were grown in three different greenhouses (S52, S55, S59) at Norwich, UK. Assayed growth parameters included **a**, complete emergence of the flag leaf (Zadoks growth stage 47), **b**, first emergence of awns (Zadok scale 49), **c**, one-quarter emergence of spike (Zadoks growth stage 53), **d**, total number of seeds harvested, and **e**, 1,000 grain weight. **a-d**, Significant differences based on ANOVA using factors genotype and location with adjusted p-values and the Tukey Honest Significant Difference test. **e**, Significant differences based on pair-wise Wilcoxon rank sum tests using adjusted p-values and the Benjamini-Hochberg approach. Significance shown with * and ** indicates adjusted $0.01 < p < 0.05$ and $0.001 < p \leq 0.01$, respectively.

Supplementary Table 1 | Infection types for wheat lines carrying and lacking *SuSr-D1* that were inoculated with diverse *Pgt* races.

		Wheat line[a]					
***Pgt* race**	**Origin**	**CTH**	**CTH 7DL**	**CTH 7DS**	**NS1**	**NS2**	**Effective/Ineffective *Sr* genes**
QTHJC (C25)	Canada	33^{+}	33^{-}	0;	0;	0;	7b,9a,9e,24,30,31,36,38,Tmp/5,6,8a,9b,9d,9g,10,11,17,21,McN
TPLKC (C33)	Canada	33^{+}	33^{-}	$0;1^{-}$	0;	0;	6,9a,9b,17,24,30,31,38/5,7b,8a,9d,9e,9g,10,11,21,36,McN,Tmp
TPMKC (C53)	Canada	33^{+}	3^{+}	;1	0;	0;	6,9a,9b,24,30,31,38/5,7b,8a,9d,9e,9g,10,11,17,21,36,McN,Tmp
QCCSC (C56)	Canada	$;2^{+}$	$;2^{-}$	0;	0;	0;	6,7b,8a,9b,9e,11,24,30,31,36,38,Tmp/5,9g,9a,9d,9g,10,17,21,McN
RTHJF (C57)	Canada	$3^{+}4$	33^{+}	$;1^{+}$	0	0	9a,9e,24,30,31,36,Tmp/5,6,7b,8a,9b,9d,9g,10,11,17,21,38,McN
SPMMC (C74)	Canada	34	33^{+}	;1	$;1^{-}$	0;	6,7b,9b,9d,10,24,30,31,38/5,8a,9a,9e,9g,11,17,21,36,McN,Tmp
RKQQC (C35)	Canada	33^{-}	$2^{+}3^{-}$	;1	;1-	0;	9e,10,11,17,24,30,31,38,Tmp/5,6,7b,8a,9a,9b,9d,9g,21,36,McN
MCCFC (C17)	Canada	$;1^{-}$	$;1^{-}$	0;	0	0	6,8a,9a,9b,9d,9e,11,21,24,30,31,36,38/5,7b,9g,10,17,McN,Tmp
QFCSC	USA	0;	0;	0	0	0	6,7b,9b,9e,11,24,30,31,36,38,Tmp/5,8a,9a,9d,9g,10,17,21,McN
QTHJC	USA	23;	$2^{+}3^{-}$	0;	0	0	7b,9a,9e,24,30,31,36,38,Tmp/5,6,8a,9b,9d,9g,10,11,17,21,McN
MCCFC	USA	;	;	N.t.	;	;	6,8a,9a,9b,9d,9e,11,21,24,30,31,36,38/5,7b,9g,10,17,McN,Tmp
TTKSK	Kenya	$3^{-}1;$	3	;1	;1	$;13^{-}$	24,36,Tmp/5,6,7b,8a,9a,9b,9d,9e,9g,10,11,17,21,30,31,38,McN
TTKSK[b]	Kenya	33^{+}	N.t.	N.t.	$2^{+}3;$	$2^{+}3;$	24,36,Tmp/5,6,7b,8a,9a,9b,9d,9e,9g,10,11,17,21,30,31,38,McN
TTKST	Kenya	31;	3	$;13^{-}$	;1	$;11^{+}$	36,Tmp/5,6,7b,8a,9a,9b,9d,9e,9g,10,11,17,21,24,30,31,38,McN
TTTSK	Kenya	$3^{-};$	3;	$;13^{-}$	$;11^{+}$	;1	24,Tmp/5,6,7b,8a,9a,9b,9d,9e,9g,10,11,17,21,30,31,36,38,McN
TRTTF	Yemen	3^{+}	3	$2^{+}3^{-}$	3	3^{+}	8a,24,31/5,6,7b,9a,9b,9d,9e,9g,10,11,17,21,30,36,38,McN,Tmp
TPMKC	USA	3;	$3^{+};$	$;13^{-}$	$;1^{+}$	$;11^{+}$	6,9a,9b,24,30,31,38/5,7b,8a,9d,9e,9g,10,11,17,21,36,McN,Tmp
21-2,5	Australia	$0;^{=}$	$0;^{=}$	N.t.	$0;^{=}$	$0;^{-}$	5,6,8a,9e,13,14,21,24,26,27,30,31,36,38,Tmp/ 7b,9d,9g,11,16,17,18,19,20,McN
34-1,2,3,5,6,7	Australia	3^{+}	3^{+}	N.t.	;12	;1	9e,13,14,21,24,26,27,30,31,36,38,Tmp/ 5,6,7b,8a, 9a,9b,9d,9g,11,15,16,17,18,19,20,28,McN

[a]N.t. refers to lines that were not tested.

[b]*Pgt* race TTKSK was tested twice. The infection types listed here are represented in Fig. 1.

Supplementary Table 2 | Adult plant disease ratings of CTH and mutant derivatives in field tests in Kenya inoculated with *Pgt* races from the (Ug99 race group.

	Field stem rust ratings[a]											
**Year**	**CTH-K**	**NS1**	**NS2**	**CTH-W**	**W01**	**W02**	**W03**	**W06**	**W07**	**W10**	**W11**	**W12**
2009	40 MS	10 M	10 M	50 MS-S	10 MR	10 MR	5 MR	5 R-MR	10 MR	10 MR	20 M	20 M
2010	15 MR	5 R	5 R	15M	5R	5R	5RMR	5R	1R	5MR	10M	10RMR
2011	20 MS	10 M	10 M	-[b]	-	-	-	-	-	-	-	-
2012	10 M	5 MR	5 MR	-	-	-	-	-	-	-	-	-
2016	20 MS	0	0	-	-	-	-	-	-	-	-	-

[a]Ratings with disease severity and infection response are based on a modified Cobb scale [44].

[b]Indicates that the line was not tested.

Supplementary Table 3 | Purity and total DNA amounts obtained after amplification of DNA from flow sorted chromosomes/arms of wild-type and mutant accessions.

Source of chromosome/arm	Purity[a] (%)	µg
Canthatch K 7D	62	11.30
Canthatch M NS1 7D	75	11.38
Canthatch M NS2 7D	77	8.09
Canthatch 7DL	85	8.26
Thatcher 7D	65	8.13
Canthatch W 7D	60	9.75
Canthatch W01 7D	55	7.90
Canthatch W02 7D	61	5.41
Canthatch W06 7D	64	6.57
Canthatch W07 7D	59	7.90
Canthatch W10 7D	61	11.17
Canthatch W11 7D	65	10.78

[a]Primary contamination from chromosome 2D.

Supplementary Table 4 | Annotated genes in the *SuSr-D1* interval.

Gene/Marker	Confidence	Start[a]	Stop	Annotation
TraesCS7D01G525800	HC	23114	27982	NB-LRR[b]
TraesCS7D01G711200	LC	32665	33072	SU(VAR)3-9 homolog
TraesCS7D01G525900	HC	40413	43559	Two-component response regulator
TraesCS7D01G526000	HC	212322	216701	Transcription factor GTE12
TraesCS7D01G711300	LC	344948	346698	RLK[c]
TraesCS7D01G526100	HC	368578	379189	Expressed[d], *Med15*
TraesCS7D01G526200	HC	388600	394682	Receptor-like protein kinase
TraesCS7D01G526300	HC	462239	469317	NB-LRR
TraesCS7D01G711400	LC	506928	507275	BED zinc finger
TraesCS7D01G711500	LC	533958	535641	NADH-cytochrome b5 reductase
TraesCS7D01G526400	HC	540932	548081	RLK
TraesCS7D01G711600	LC	549339	555565	Coatomer subunit beta
TraesCS7D01G526500	HC	596661	609536	TraesCS7D01G526500
TraesCS7D01G711700	LC	684459	685613	LINE-1 reverse transcriptase
TraesCS7D01G711800	LC	686316	686810	Retrotransposon protein
TraesCS7D01G526600	HC	698149	702833	NB-LRR
TraesCS7D01G526700	HC	715220	717587	Expressed, RLK
TraesCS7D01G711900	LC	718856	727423	Transposon protein
TraesCS7D01G526800	HC	731531	733548	Expressed, RLK
TraesCS7D01G712000	LC	736447	737076	FAR1-related sequence
TraesCS7D01G526900	HC	743932	744540	F-box-like/WD repeat-containing protein
TraesCS7D01G712100	LC	747998	748713	Serine/threonine-protein phosphatase
TraesCS7D01G712200	LC	819678	820382	Zinc finger MYM-type-like protein
TraesCS7D01G712300	LC	821274	822360	Zinc finger MYM-type-like protein
TraesCS7D01G527000	HC	893274	905402	RLK
TraesCS7D01G527100	HC	916387	917770	RLK
TraesCS7D01G527200	HC	945407	951862	NB-LRR
TraesCS7D01G527300	HC	959904	962258	Ubiquitin carboxyl-terminal hydrolase
TraesCS7D01G527400	HC	1000682	1002679	FAR1-related sequence

[a]Coordinates in base pairs from a start position using marker *kwh239*.
[b]Nucleotide-binding, leucine-rich repeat protein.
[c]Receptor-like kinase.
[d]Expression based on RNAseq of first and second leaf tissue of CTH and *SuSr-D1* mutants NS1 and NS2.

Supplementary Table 5 | SNVs identified between CTH and mutants NS1 and NS2 based on strict and relaxed parameter selection.

		**Strict**		**Relaxed**	
**Transition**	**Type**	**CTH vs. NS1**	**CTH vs. NS2**	**CTH vs. NS1**	**CTH vs. NS2**
A → C	Background	10	7	403	180
A → G	Background	41	15	1,321	592
A → T	Background	7	4	285	121
C → A	Background	4	4	211	87
C → G	Background	4	3	295	129
C → T	EMS	107	124	799	473
G → A	EMS	103	131	727	498
G → C	Background	11	5	276	131
G → T	Background	8	4	215	91
T → A	Background	10	6	247	113
T → C	Background	30	13	1,316	596
T → G	Background	10	7	411	185
Subtotal	EMS	210	255	1,526	971
**Total**	All	345	323	6,506	3,196

Supplementary Table 6 | Primer sequences for SNV markers used to map *SuSr-D1* in the Thatcher x NS1 and Thatcher x NS2 DH populations.

		Primer Sequences[a]		
Marker	Population	Allele-specific primer 1	Allele-specific primer 2	Common primer
kwh196	NS1	AGTGCAGAAGAAGAAAAAAA	AGTGCAGAAGAAGAAAAAAG	ACAGTGCCAGTTGTCCGG
kwh198	NS1	ATTAACACCGGTTAAGGTTA	ATTAACACCGGTTAAGGTTG	GGGTGAGCTGTAGTGCAGG
kwh200	NS1	ATTGATGACTTTGATTATGA	ATTGATGACTTTGATTATGG	TCAGTTGCTCTCGCTGTGG
kwh202	NS1	GCCGCCGACAAGCATCCCGC	GCCGCCGACAAGCATCCCGT	TCGCGTCGACGGAAAACA
kwh203	NS1	GGCAGAGTCATGCAGCAAGC	GGCAGAGTCATGCAGCAAGT	CTATCCCGCAGCTTCCGG
kwh204	NS1	TGCTGTTGTTGCTGCGTTTA	TGCTGTTGTTGCTGCGTTTG	ACAGCAGCAGCAACAAAATCA
kwh205	NS1	ATGAAGATCGCGGAGAAATA	ATGAAGATCGCGGAGAAATG	TCGTGCCTCTCGACCTCA
kwh213	NS2	GGACTTAGCAATGGCGACGC	GGACTTAGCAATGGCGACGT	CCCTTGAGCTGCACGACA
kwh214	NS2	ATATCGCTGATAGTGGCCCC	ATATCGCTGATAGTGGCCCT	CTCTAGCTCGCCCCTCCA
kwh215	NS2	CAGCACAAACACCAACACGA	CAGCACAAACACCAACACGG	TGGTGACAGTCTGGCAGG
kwh216	NS2	GCGACCATTATCATCCATAA	GCGACCATTATCATCCATAG	CTCCAGGTGCTACGACGTC
kwh235	NS1	GATTGCATCACAGTTGCTGC	GATTGCATCACAGTTGCTGT	ATCGGATGCTGGCGGATG
kwh236	NS1	AGATCACGTCTGACATTGGC	AGATCACGTCTGACATTGGT	TTTTTCGCGCCGCTATGC
kwh239	NS1	ATTCTCTCCAGAGATGTCGC	ATTCTCTCCAGAGATGTCGT	GTGCCTTGTTCTTGCGTGA
kwh243	NS2	TCCACAGTATTTTCAATGGC	TCCACAGTATTTTCAATGGT	CGTGCATGTCATTGATTCCCA
kwh245	NS2	ATCTCTCCGGAACATCCAAC	ATCTCTCCGGAACATCCAAT	GCCTCCATTGCTGCAGGA
kwh246	NS2	CCAGTTATGAAAATCAAGAA	CCAGTTATGAAAATCAAGAG	TCGTACGACACCTAGTTCACT
kwh247	NS2	GAGCTAACCATATCTAATGC	GAGCTAACCATATCTAATGT	CCACGATCTTGCATGTGCG
kwh252	NS1	ACTACGAGAAGCAAAATAAA	ACTACGAGAAGCAAAATAAG	TCTGATCCAAGCTTCCTTGCT
kwh255	NS2	AACAATTTGTGTAAAACAAA	AACAATTTGTGTAAAACAAG	TGCTTTTCGGGCTGCCAT
kwh261	NS2	TTTAAGCTGGGGAGAGGCAA	TTTAAGCTGGGGAGAGGCAG	GGCAAGTTCTTTAGGGCAGC
kwh264	NS2	GAAGCGTCAGATGTTTCAGC	GAAGCGTCAGATGTTTCAGT	TGCTGTATTGGGAACTGCTGT
kwh265	NS1	GCAGGATTGGTGAACTGACA	GCAGGATTGGTGAACTGACG	GACGATGTCCTTCCCGCC
kwh266	NS2	AATGAAATTGGGGAGATATC	AATGAAATTGGGGAGATATT	CGCAACCCAACCTAATCTACT
kwh267	NS2	TTTATTTGTTGAAGAAAGCA	TTTATTTGTTGAAGAAAGCG	TGGAACGGAGGGAGTAGAACT
kwh271	NS2	GTATCATTGTATTTGTGTTA	GTATCATTGTATTTGTGTTG	CTGGTGTTCGTTGTACTGACA
kwh275	NS2	CCCATCGAACAGTACCCCGA	CCCATCGAACAGTACCCCGG	CGTCCAGCGCCTCTTCAG
kwh278	NS2	GGGTACTTTTACCTTCAAAA	GGGTACTTTTACCTTCAAAG	CGGTGGCTCTAGCTCTGC
kwh281	NS1	AGTGGTAGCGTGTGCATGCA	AGTGGTAGCGTGTGCATGCG	TACACAGCACCACGGCTG
kwh285	NS1	TCGCCCTATGAAGAGGCCAA	TCGCCCTATGAAGAGGCCAG	TTACGCCCGTGCTAGTCG
kwh286	NS1	ACCTCAAAACGGCCTCCTGA	ACCTCAAAACGGCCTCCTGG	AGCGCCCCTTCTTCTTTTCA
kwh289	NS1	TTTGGCTGAAAAAAATCAAA	TTTGGCTGAAAAAAATCAAG	ATGGTCTGGGCCTTTGCG
kwh295	NS1	GTCGCCCTCGGCTTGTTCGC	GTCGCCCTCGGCTTGTTCGT	ACCGCTAAGCACGTGTCC
kwh297	NS2	GGGCAGCTTAAAAATTAAGC	GGGCAGCTTAAAAATTAAGT	TTAAGCTGGGGAGAGGCA
kwh299	NS2	GAAGCGTCAGATGTTTCAGC	GAAGCGTCAGATGTTTCAGT	TGCTGTATTGGGAACTGCTGT

[a]Allele-specific amplification requires the 5` tail sequence provided by LGC Genomics (UK).

Supplementary Table 7 | Physical locations of SNV markers on the IWGSC RefSeq Ver 1.0 assembly of wheat chromosome 7D.

Marker[a]	Population	Physical position (bp)
kwh196	NS1	411,304,393
kwh198	NS1	461,144,282
kwh200	NS1	501,096,332
kwh213	NS2	530,923,382
kwh214	NS2	554,332,418
kwh202	NS1	571,190,062
kwh215	NS2	571,257,742
kwh203	NS1	593,178,556
kwh216	NS2	597,472,194
kwh243	NS2	616,366,834
kwh236	NS1	616,954,328
kwh245	NS2	617,054,080
kwh235	NS1	617,087,147
kwh246	NS2	617,601,546
kwh255	NS2	620,471,122
kwh252	NS1	620,575,636
kwh247	NS2	621,071,925
kwh239	NS1	621,270,682
kwh297	NS2	621,640,467
kwh261	NS2	621,640,505
kwh295	NS1	621,643,223
kwh204	NS1	621,644,915
kwh264	NS2	621,647,501
kwh299	NS2	621,647,501
kwh265	NS1	621,733,638
kwh266	NS2	621,819,606
kwh267	NS2	621,819,644
kwh281	NS1	622,350,270
kwh285	NS1	626,550,873
kwh271	NS2	626,753,797
kwh275	NS2	627,214,114
kwh286	NS1	628,218,276
kwh278	NS2	631,343,087
kwh289	NS1	631,858,504
kwh205	NS1	635,581,069

[a]Markers in italics detect SNVs within the *SuSr-D1* sequence.

Supplementary Table 8 | Gene identifiers of the *Med15* gene family in diverse species.

**Species**	**Gene**	**Chromosome/arm**	**Identifier**
Brachypodium distachyon	Bradi3g12660.1	Bd3	*BdMed15*
Hordeum vulgare	HORVU7Hr1G060970.1	7HS	*HvMed15a*
Hordeum vulgare	HORVU7Hr1G118370.1	7HL	*HvMed15b*
Oryza sativa	Os08g45080.1	Os8	*OsMed15*
Setaria italica	Seita.6G253900.2	Si6	*SiMed15*
Sorghum bicolor	Sobic.007G163100.2	Sb7	*SbMed15*
Triticum aestivum	TraesCS7A01G283500.3	7AS	*TaMed15a.A*
Triticum aestivum	TraesCS7A01G540000.1	7AL	*TaMed15b.A*
Triticum aestivum	TraesCS7B01G177500.1	7BS	*TaMed15a.B*
Triticum aestivum	TraesCS7B01G460900.1	7BL	*TaMed15b.B*
Triticum aestivum	TraesCS7D01G281700.2	7DS	*TaMed15a.D*
Triticum aestivum	TraesCS7D01G526100.1	7DL	*TaMed15b.D*

Supplementary Table 9 | Parameter and log likelihood estimates of the rate of protein evolution among *Med15* homologs.

**Model**	**κ**	**ω_0**	**ω_1**	**ω_a**	**ω_b**	**lnL**
H_0 (one ω)	2.872	0.289	$= \omega_0$	$= \omega_0$	$= \omega_0$	-12542.10
H_1 (two ω; ω_0 and ω_1)	2.873	0.288	0.310	$= \omega_0$	$= \omega_0$	-12542.08
H_2 (two ω; ω_0 and ω_b)	2.874	0.269	$= \omega_0$	$= \omega_0$	0.372	-12538.56
H_3 (three ω; ω_0, ω_a, ω_b)	2.879	0.244	$= \omega_0$	0.377	0.372	-12533.71

Significant differences in ω were observed for H_0 vs. H_2 (p=0.007) and H_2 vs. H_3 (p=0.002). Variables: κ, transition:transversion ratio, ω d_N/d_S ratio, lnL, log-likelihood. All likelihood ratio tests had one degree of freedom.

Supplementary Table 10 | Differentially expressed genes between wild-type and mutant *SuSr-D1* in non-inoculated plants.

	Wild-type versus *SuSr-D1-NS1*			Wild-type versus *SuSr-D1-NS2*			
**Gene**	**Fold change (log10)**	***p*-value**	***q*-value**	**Fold change (log10)**	***p*-value**	***q*-value**	**Description**
TraesCS1A01G153100.3	0.282	7.4E-05	1.4E-02	0.280	7.6E-05	2.7E-02	Long-chain-alcohol oxidase
TraesCS1A01G350500.1	0.265	6.7E-08	2.0E-05	0.266	6.4E-08	3.7E-05	Non-specific serine/threonine protein kinase
TraesCS1A01G352500.1	0.388	3.0E-08	9.2E-06	0.389	2.9E-08	1.7E-05	Acid phosphatase/vanadium-dependent haloperoxidase-related
TraesCS1A01G353300.1	0.212	1.3E-05	2.9E-03	0.264	7.1E-08	4.1E-05	Glycogen synthase
TraesCS1A01G353800.1	0.285	1.2E-07	3.5E-05	0.298	3.3E-08	1.9E-05	Serine acetyltransferase
TraesCS1A01G357500.3	0.240	4.4E-05	8.8E-03	0.248	2.4E-05	9.7E-03	IQ domain-containing protein
TraesCS1A01G358000.1	0.201	2.7E-04	4.6E-02	0.222	6.5E-05	2.4E-02	Ras-like protein
TraesCS1A01G359300.1	0.240	2.9E-05	6.2E-03	0.293	4.0E-07	2.2E-04	Alpha/beta-Hydrolases superfamily protein
TraesCS1A01G360500.2	0.226	8.0E-06	1.8E-03	0.270	1.0E-07	5.9E-05	Nucleosome assembly protein 1-like 1
TraesCS1A01G362000.1	0.261	2.3E-05	5.0E-03	0.276	8.3E-06	3.7E-03	Histone-lysine N-methyltransferase, H3 lysine-9 specific
TraesCS1A01G364500.1	0.204	8.8E-05	1.6E-02	0.219	2.7E-05	1.1E-02	Saposin B domain protein
TraesCS1A01G367300.1	0.279	5.6E-06	1.3E-03	0.274	8.4E-06	3.7E-03	Transcription factor GTE1
TraesCS1A01G368000.1	0.327	6.7E-12	2.5E-09	0.315	4.0E-11	3.0E-08	Histone H2B
TraesCS1A01G368100.1	0.300	2.1E-08	6.4E-06	0.366	1.2E-11	8.9E-09	DNA-binding
TraesCS1A01G369900.1	0.294	4.4E-05	9.0E-03	0.466	3.7E-10	2.5E-07	Splicing factor 3B subunit 5
TraesCS1A01G370800.2	0.229	4.2E-05	8.5E-03	0.263	2.8E-06	1.4E-03	Hexosyltransferase
TraesCS1A01G373000.1	0.266	9.1E-07	2.4E-04	0.251	3.8E-06	1.8E-03	Lipid phosphate phosphatase-like protein
TraesCS1A01G374200.1	0.306	1.6E-07	4.4E-05	0.298	3.3E-07	1.9E-04	Lipid transfer protein
TraesCS1A01G375800.1	0.215	5.7E-05	1.1E-02	0.237	8.9E-06	3.9E-03	1,2-dihydroxy-3-keto-5-methylthiopentene dioxygenase
TraesCS1A01G375900.1	0.238	6.3E-05	1.2E-02	0.245	4.1E-05	1.6E-02	Thymidine kinase
TraesCS1A01G376100.1	0.246	5.7E-14	2.4E-11	0.256	6.7E-15	5.7E-12	U4/U6 small nuclear ribonucleoprotein Prp31
TraesCS1A01G378200.1	0.267	3.0E-06	7.3E-04	0.251	1.1E-05	4.7E-03	Sulfhydryl oxidase
TraesCS1A01G379000.1	0.259	4.3E-09	1.4E-06	0.300	1.2E-11	8.9E-09	ATP synthase subunit beta
TraesCS1A01G379300.1	0.223	8.7E-05	1.6E-02	0.295	2.9E-07	1.6E-04	Nucleolar family protein
TraesCS1A01G379800.1	0.285	3.9E-10	1.3E-07	0.289	2.6E-10	1.8E-07	Syntaxin, putative
TraesCS1A01G380900.1	0.258	3.0E-06	7.3E-04	0.270	1.1E-06	5.7E-04	Protein DETOXIFICATION
TraesCS1A01G381200.1	0.246	5.8E-08	1.7E-05	0.268	3.6E-09	2.3E-06	ER membrane protein complex subunit 6
TraesCS1A01G382800.1	0.234	1.0E-04	1.9E-02	0.253	3.0E-05	1.2E-02	Asparagine synthetase
TraesCS1A01G388000.1	0.302	9.6E-09	3.0E-06	0.244	3.4E-06	1.7E-03	Ubiquitin-conjugating enzyme E2
TraesCS1A01G392400.1	0.236	1.5E-06	3.8E-04	0.248	4.3E-07	2.4E-04	Ubiquitin-conjugating enzyme E2
TraesCS1A01G395200.1	0.302	1.8E-12	7.2E-10	0.280	7.1E-11	5.0E-08	Protein DEHYDRATION-INDUCED 19
TraesCS1A01G512200LC.1	0.208	6.7E-05	1.3E-02	0.251	2.0E-06	9.9E-04	Mitochondrial outer membrane porin
TraesCS1B01G141100.1	0.329	2.3E-04	4.0E-02	0.346	1.1E-04	3.7E-02	nuclease
TraesCS1B01G241900.2	-0.450	6.0E-07	1.6E-04	-0.424	2.6E-06	1.3E-03	Serine/threonine-protein kinase
TraesCS1B01G241900.3	0.441	6.6E-12	2.5E-09	0.249	9.5E-05	3.3E-02	Serine/threonine-protein kinase
TraesCS1B01G377300.1	-0.308	1.6E-08	5.0E-06	-0.311	1.2E-08	7.6E-06	Mitochondrial Rho GTPase

TraesCS1D01G069500LC.1	-0.416	3.5E-06	8.3E-04	-0.606	1.1E-11	8.7E-09	Core-2/I-branching beta-1,6-N-acetylglucosaminyltransferase
TraesCS1D01G153000LC.1	0.469	2.9E-08	8.9E-06	0.481	1.2E-08	7.5E-06	Gag polyprotein
TraesCS1D01G353300.1	-1.258	5.2E-64	9.1E-61	-1.285	8.5E-67	3.1E-63	Non-specific serine/threonine protein kinase
TraesCS1D01G353400.2	-0.414	3.5E-06	8.4E-04	-0.407	5.1E-06	2.4E-03	WD-repeat protein, putative
TraesCS1D01G353800.1	-0.371	2.8E-05	5.8E-03	-0.445	5.1E-07	2.8E-04	senescence regulator (Protein of unknown function, DUF584)
TraesCS1D01G354500.2	-0.476	1.1E-07	3.2E-05	-0.431	1.5E-06	7.7E-04	2-isopropylmalate synthase
TraesCS1D01G354900.1	-0.785	5.8E-19	3.0E-16	-0.756	1.1E-17	1.1E-14	DUF616 family protein
TraesCS1D01G355200.2	-1.410	1.2E-80	3.5E-77	-1.416	2.7E-81	1.6E-77	Phosphotransferase
TraesCS1D01G355400.1	-0.619	4.6E-12	1.8E-09	-0.574	1.5E-10	1.0E-07	Cleavage and polyadenylation specificity factor subunit 5
TraesCS1D01G355800.1	-1.743	3.3E-145	3.6E-141	-1.717	7.4E-141	2.5E-136	RNA binding protein
TraesCS1D01G355900.1	-0.413	3.4E-06	8.2E-04	-0.396	8.3E-06	3.7E-03	Transmembrane protein
TraesCS1D01G356000.1	-1.426	2.3E-74	5.3E-71	-1.383	5.2E-70	2.2E-66	TPR repeat
TraesCS1D01G356000.3	-0.640	6.3E-13	2.6E-10	-0.584	5.6E-11	4.0E-08	TPR repeat
TraesCS1D01G356800.1	-1.282	4.4E-66	8.0E-63	-1.305	1.9E-68	7.3E-65	Serine acetyltransferase
TraesCS1D01G356900.1	-1.610	6.5E-114	3.2E-110	-1.594	1.2E-111	1.5E-107	Starch synthase
TraesCS1D01G357000.1	-0.405	5.2E-06	1.2E-03	-0.432	1.2E-06	6.1E-04	Glycerophosphodiester phosphodiesterase
TraesCS1D01G357200.1	-1.127	1.4E-44	1.5E-41	-1.071	2.7E-40	5.2E-37	Mitochondrial ATP synthase g subunit family protein
TraesCS1D01G357300.1	-0.747	2.5E-17	1.2E-14	-0.706	1.3E-15	1.2E-12	ATP-dependent Clp protease ATP-binding subunit ClpX
TraesCS1D01G357400.1	-0.817	7.5E-22	4.2E-19	-0.868	1.8E-24	2.1E-21	Pentatricopeptide repeat-containing protein
TraesCS1D01G357600.1	-1.187	4.6E-50	5.6E-47	-1.217	1.4E-52	3.2E-49	Acid phosphatase/vanadium-dependent haloperoxidase-related protein
TraesCS1D01G358500.1	-0.382	1.4E-05	3.2E-03	-0.380	1.6E-05	6.6E-03	Glycosyltransferase
TraesCS1D01G360800.1	-1.269	3.7E-66	6.8E-63	-1.306	4.9E-70	2.2E-66	Pentatricopeptide repeat-containing protein, putative
TraesCS1D01G361000.1	-0.783	8.6E-19	4.5E-16	-0.791	4.0E-19	4.2E-16	Choline/ethanolamine kinase
TraesCS1D01G361100.1	-0.637	5.8E-13	2.4E-10	-0.724	2.3E-16	2.1E-13	F-box protein
TraesCS1D01G361400.1	-1.091	1.1E-41	1.1E-38	-1.026	4.8E-37	8.4E-34	6-phosphogluconate dehydrogenase, decarboxylating
TraesCS1D01G361600.1	-0.744	4.0E-27	2.7E-24	-0.723	1.2E-25	1.5E-22	ABC transporter A family protein
TraesCS1D01G361700.1	-1.039	3.4E-34	2.8E-31	-1.032	9.4E-34	1.5E-30	30S ribosomal protein S1-like
TraesCS1D01G361900.1	-0.948	1.8E-61	2.9E-58	-0.897	5.8E-55	1.5E-51	Mitochondrial outer membrane porin
TraesCS1D01G362700.1	-1.334	5.5E-68	1.1E-64	-1.299	1.8E-64	6.3E-61	Aldehyde dehydrogenase
TraesCS1D01G362800.1	-1.144	3.4E-52	4.4E-49	-1.166	4.0E-54	9.8E-51	Ras-like protein
TraesCS1D01G363500.1	-0.721	6.5E-16	2.9E-13	-0.762	1.3E-17	1.3E-14	Protein phosphatase 2C
TraesCS1D01G363700.1	-1.162	7.4E-51	9.3E-48	-1.163	7.2E-51	1.6E-47	Beta-galactosidase
TraesCS1D01G363900.2	-0.794	6.3E-20	3.4E-17	-0.793	7.5E-20	8.0E-17	Alpha/beta-Hydrolases superfamily protein
TraesCS1D01G364700.1	-0.911	1.1E-28	7.9E-26	-0.868	4.9E-26	6.1E-23	Phosphoribosylformylglycinamidine synthase family protein
TraesCS1D01G364800.1	-0.877	6.8E-25	4.3E-22	-0.938	2.7E-28	3.6E-25	Mitochondrial carrier family
TraesCS1D01G364900.1	-1.358	7.9E-74	1.7E-70	-1.351	5.2E-73	2.5E-69	Nucleosome assembly protein 1-like 1
TraesCS1D01G365400.1	-1.463	2.1E-102	8.1E-99	-1.492	1.4E-106	1.6E-102	DUF1230 family protein
TraesCS1D01G365500.1	-1.102	3.8E-38	3.4E-35	-1.079	1.2E-36	2.1E-33	Exostosin-2
TraesCS1D01G365600.1	-0.628	1.2E-12	4.9E-10	-0.661	6.8E-14	5.6E-11	Flap endonuclease 1
TraesCS1D01G365700.1	-0.491	4.7E-10	1.6E-07	-0.508	1.2E-10	8.4E-08	Embryo defective 2752 protein
TraesCS1D01G366200.1	-0.950	1.4E-29	1.0E-26	-0.942	4.5E-29	6.1E-26	SWR1-complex protein 4/DNA methyltransferase 1-associated protein 1

TraesCS1D01G366800.1	-0.856	5.9E-23	3.4E-20	-0.902	2.6E-25	3.2E-22	Histone-lysine N-methyltransferase, H3 lysine-9 specific
TraesCS1D01G367000.1	-0.844	1.6E-21	9.2E-19	-0.827	9.8E-21	1.1E-17	cotton fiber-like protein (DUF761)
TraesCS1D01G367100.1	-0.371	2.6E-05	5.5E-03	-0.414	2.7E-06	1.4E-03	Microtubule associated protein 65
TraesCS1D01G367200.1	-0.580	1.2E-10	4.1E-08	-0.600	2.7E-11	2.0E-08	F-box family protein
TraesCS1D01G367600.1	-1.685	1.0E-113	4.8E-110	-1.618	6.9E-105	7.1E-101	Homogentisate phytyltransferase
TraesCS1D01G367700.1	-0.346	9.0E-05	1.7E-02	-0.397	7.1E-06	3.2E-03	basic helix-loop-helix (bHLH) DNA-binding superfamily protein
TraesCS1D01G368100.1	-1.015	5.6E-33	4.4E-30	-1.054	2.0E-35	3.4E-32	Vesicle transport V-SNARE protein
TraesCS1D01G368200.1	-0.508	1.6E-08	5.0E-06	-0.617	7.1E-12	5.5E-09	Vesicle transport v-SNARE family protein
TraesCS1D01G368300.1	-1.261	1.4E-63	2.5E-60	-1.303	7.7E-68	2.9E-64	DUF1639 family protein
TraesCS1D01G368700.1	-1.029	9.1E-34	7.3E-31	-0.928	1.1E-27	1.4E-24	60S ribosomal protein l28
TraesCS1D01G368800.1	-1.275	2.0E-62	3.3E-59	-1.215	1.1E-56	3.0E-53	Fiber protein Fb11
TraesCS1D01G368900.1	-1.056	7.3E-35	6.3E-32	-0.986	1.6E-30	2.3E-27	Alpha/beta-hydrolase
TraesCS1D01G368900.2	-1.084	3.1E-38	2.8E-35	-1.060	1.4E-36	2.4E-33	Alpha/beta-hydrolase
TraesCS1D01G369600.1	-1.176	1.8E-55	2.6E-52	-1.229	1.6E-60	5.1E-57	StAR-related lipid transfer protein
TraesCS1D01G369600.2	-1.332	2.5E-65	4.4E-62	-1.302	2.2E-62	7.5E-59	StAR-related lipid transfer protein
TraesCS1D01G369900.1	-1.048	2.7E-39	2.6E-36	-1.123	4.7E-45	9.8E-42	Saposin B domain protein
TraesCS1D01G370100.1	-0.648	4.0E-14	1.7E-11	-0.764	4.0E-19	4.2E-16	Plant/F18B13-26 protein
TraesCS1D01G370300.3	-0.693	1.4E-14	6.3E-12	-0.698	8.8E-15	7.4E-12	Pectin lyase-like superfamily protein
TraesCS1D01G370600.1	-0.945	1.4E-34	1.1E-31	-0.873	1.2E-29	1.7E-26	Phospholipase D
TraesCS1D01G370900.1	-0.454	4.3E-07	1.2E-04	-0.512	1.2E-08	7.5E-06	Glucuronoxylan 4-O-methyltransferase
TraesCS1D01G371000.1	-0.599	3.2E-12	1.2E-09	-0.676	3.1E-15	2.7E-12	AP-1 complex subunit mu-1
TraesCS1D01G371000.2	-0.555	6.9E-10	2.3E-07	-0.538	2.3E-09	1.5E-06	AP-1 complex subunit mu-1
TraesCS1D01G371100.1	-0.524	5.9E-09	1.9E-06	-0.546	1.3E-09	8.8E-07	WD repeat protein-like
TraesCS1D01G371200.1	-0.835	4.1E-23	2.4E-20	-0.819	3.1E-22	3.4E-19	Farnesyl pyrophosphate synthase
TraesCS1D01G371400.1	-0.395	6.8E-06	1.6E-03	-0.358	4.5E-05	1.7E-02	MYB transcription factor
TraesCS1D01G371700.1	-0.502	2.6E-08	7.8E-06	-0.460	3.2E-07	1.8E-04	MYB transcription factor
TraesCS1D01G371800.1	-0.371	2.2E-05	4.8E-03	-0.334	1.3E-04	4.5E-02	MYB transcription factor
TraesCS1D01G372000.1	-1.133	1.9E-68	3.9E-65	-1.235	2.9E-81	1.6E-77	Chaperone protein dnaJ, putative
TraesCS1D01G372100.1	-1.123	8.4E-46	9.4E-43	-1.223	2.8E-54	6.9E-51	Cytochrome P450, putative
TraesCS1D01G372200.1	-1.175	1.4E-50	1.7E-47	-1.230	2.3E-55	6.1E-52	Sec14p-like phosphatidylinositol transfer family protein
TraesCS1D01G372200.2	-1.678	8.5E-118	4.6E-114	-1.726	1.7E-124	2.5E-120	Sec14p-like phosphatidylinositol transfer family protein
TraesCS1D01G372200.3	-0.399	9.2E-06	2.1E-03	-0.372	3.6E-05	1.4E-02	Sec14p-like phosphatidylinositol transfer family protein
TraesCS1D01G372300.1	-0.963	3.8E-31	3.0E-28	-0.937	1.7E-29	2.4E-26	Regulator of chromosome condensation (RCC1) family protein
TraesCS1D01G373000.1	-0.715	3.3E-16	1.5E-13	-0.732	6.7E-17	6.4E-14	Transcription factor GTE1
TraesCS1D01G373800.1	-0.749	2.3E-18	1.2E-15	-0.857	1.2E-23	1.4E-20	Histone H2B
TraesCS1D01G374000.1	-1.132	4.3E-45	4.7E-42	-1.134	3.2E-45	6.8E-42	DNA-binding
TraesCS1D01G374100.1	-0.748	1.4E-43	1.5E-40	-0.718	3.2E-40	6.1E-37	VIP1 protein
TraesCS1D01G374300.1	-0.697	4.3E-17	2.1E-14	-0.669	9.2E-16	8.2E-13	ABC transporter B family protein
TraesCS1D01G374500.1	-0.361	5.2E-05	1.0E-02	-0.388	1.3E-05	5.6E-03	Pentatricopeptide repeat-containing protein
TraesCS1D01G374600.1	-0.874	7.0E-24	4.3E-21	-0.850	1.2E-22	1.3E-19	Acid phosphatase/vanadium-dependent haloperoxidase related
TraesCS1D01G375100.1	-0.345	7.2E-05	1.4E-02	-0.403	3.5E-06	1.7E-03	Chlorophyll a-b binding protein, chloroplastic

TraesCS1D01G375300.1	-1.010	1.6E-35	1.4E-32	-0.983	1.2E-33	2.0E-30	Glutamate--tRNA ligase
TraesCS1D01G375700.1	-1.360	2.0E-58	3.1E-55	-1.289	1.1E-52	2.5E-49	Methyltransferase
TraesCS1D01G375800.1	-1.283	8.1E-62	1.3E-58	-1.282	1.2E-61	3.9E-58	ENTH/ANTH/VHS superfamily protein
TraesCS1D01G376000.1	-2.028	3.2E-239	1.0E-234	-2.078	4.6E-251	2.4E-246	Kinase family protein
TraesCS1D01G376100.1	-0.879	3.1E-25	2.0E-22	-0.899	2.6E-26	3.3E-23	Splicing factor 3B subunit 5
TraesCS1D01G376200.1	-0.342	1.0E-04	1.9E-02	-0.388	1.1E-05	4.6E-03	(R)-specific enoyl-CoA hydratase
TraesCS1D01G377900.1	-0.615	7.4E-12	2.8E-09	-0.536	2.5E-09	1.6E-06	Purple acid phosphatase
TraesCS1D01G378000.1	-0.395	7.0E-06	1.6E-03	-0.351	6.5E-05	2.4E-02	Valine--tRNA ligase
TraesCS1D01G378000.2	-0.790	1.5E-19	8.1E-17	-0.730	7.5E-17	7.0E-14	Valine--tRNA ligase
TraesCS1D01G378100.1	-0.947	2.6E-30	2.0E-27	-0.963	2.7E-31	4.2E-28	Disease resistance protein (CC-NBS-LRR class) family
TraesCS1D01G378200.1	-1.568	2.0E-107	8.3E-104	-1.532	1.7E-102	1.6E-98	Protein phosphatase 2c, putative
TraesCS1D01G378600.1	-0.663	1.4E-13	6.0E-11	-0.678	4.2E-14	3.5E-11	Threonine synthase
TraesCS1D01G378700.1	-0.573	7.2E-13	2.9E-10	-0.572	8.1E-13	6.3E-10	Peptidyl-tRNA hydrolase
TraesCS1D01G378900.1	-0.361	2.8E-05	5.8E-03	-0.343	6.9E-05	2.5E-02	AP2/B3 transcription factor family protein
TraesCS1D01G379000.2	-1.277	3.2E-58	4.9E-55	-1.227	8.3E-54	2.0E-50	Lactation elevated protein 1
TraesCS1D01G379500.2	-0.847	3.3E-23	1.9E-20	-0.869	2.5E-24	3.0E-21	Ankyrin repeat-containing protein
TraesCS1D01G379600.1	-0.847	1.4E-23	8.6E-21	-0.959	5.8E-30	8.4E-27	CC-NBS-LRR disease resistance protein
TraesCS1D01G379800.1	-1.305	1.2E-85	3.7E-82	-1.345	6.4E-91	4.3E-87	Lipid phosphate phosphatase-like protein
TraesCS1D01G380000.2	-1.432	2.5E-79	6.9E-76	-1.487	1.7E-85	1.0E-81	RING/U-box superfamily protein
TraesCS1D01G380200.1	-1.097	1.0E-43	1.1E-40	-1.113	5.3E-45	1.1E-41	Serine/threonine-protein phosphatase
TraesCS1D01G380800.1	-1.157	1.8E-49	2.1E-46	-1.281	2.1E-60	6.3E-57	Lipid transfer protein
TraesCS1D01G380900.1	-0.608	9.8E-12	3.6E-09	-0.709	1.9E-15	1.6E-12	Receptor protein kinase, putative
TraesCS1D01G381100.1	-0.362	4.5E-05	9.1E-03	-0.487	4.4E-08	2.6E-05	Lipid transfer protein
TraesCS1D01G381400.1	-0.730	1.4E-16	6.6E-14	-0.783	7.6E-19	7.8E-16	Mitogen-activated protein kinase
TraesCS1D01G381500.1	-0.935	1.4E-29	1.0E-26	-0.952	1.3E-30	1.9E-27	Disease resistance protein (NBS-LRR class) family
TraesCS1D01G381500.2	-0.873	1.2E-23	7.5E-21	-0.836	9.4E-22	1.0E-18	Disease resistance protein (NBS-LRR class) family
TraesCS1D01G382000.1	-1.540	1.1E-98	4.2E-95	-1.556	9.5E-101	8.1E-97	Zinc finger protein-like
TraesCS1D01G382400.1	-1.511	3.8E-85	1.1E-81	-1.622	8.5E-98	6.7E-94	Transporter-like protein
TraesCS1D01G382900.1	-0.527	3.2E-09	1.0E-06	-0.337	1.5E-04	4.9E-02	Heat shock transcription factor
TraesCS1D01G383000.1	-0.537	2.5E-09	7.9E-07	-0.548	1.1E-09	7.3E-07	Membrane protein insertase YidC
TraesCS1D01G383100.1	-1.664	2.7E-129	1.7E-125	-1.638	3.5E-125	5.9E-121	1,2-dihydroxy-3-keto-5-methylthiopentene dioxygenase
TraesCS1D01G383400.2	-0.873	5.5E-36	4.9E-33	-0.897	6.9E-38	1.3E-34	U4/U6 small nuclear ribonucleoprotein Prp31
TraesCS1D01G383500.1	-1.509	1.6E-90	5.3E-87	-1.544	1.1E-94	7.9E-91	Chloroplastic group IIA intron splicing facilitator CRS1, chloroplastic
TraesCS1D01G383600.1	-0.780	1.3E-18	6.5E-16	-0.766	5.4E-18	5.5E-15	tRNA dimethylallyltransferase
TraesCS1D01G384300.1	-0.388	1.1E-05	2.4E-03	-0.349	7.0E-05	2.5E-02	Cyclopropane-fatty-acyl-phospholipid synthase
TraesCS1D01G384700.1	-0.714	6.1E-17	2.9E-14	-0.703	2.0E-16	1.9E-13	General transcription factor 3C polypeptide 5
TraesCS1D01G384900.1	-0.533	3.3E-09	1.0E-06	-0.553	8.4E-10	5.6E-07	Hexosyltransferase
TraesCS1D01G385000.1	-0.988	2.3E-31	1.8E-28	-0.948	6.1E-29	8.2E-26	Guanine nucleotide-binding protein beta subunit-like protein
TraesCS1D01G385200.1	-1.313	7.5E-67	1.4E-63	-1.330	1.5E-68	6.0E-65	Sulfhydryl oxidase
TraesCS1D01G385500.1	-1.032	1.5E-39	1.4E-36	-1.005	1.3E-37	2.4E-34	Oxygen-independent coproporphyrinogen-III oxidase-like protein
TraesCS1D01G386500.1	-1.018	4.1E-34	3.3E-31	-0.967	7.3E-31	1.1E-27	Nucleolar family protein

TraesCS1D01G386600.1	-0.565	2.8E-10	9.9E-08	-0.590	4.5E-11	3.3E-08	F-box protein
TraesCS1D01G386700.2	-0.547	8.4E-10	2.8E-07	-0.573	1.2E-10	8.5E-08	F-box protein
TraesCS1D01G387100.1	-0.361	4.2E-05	8.5E-03	-0.386	1.2E-05	5.0E-03	Myb transcription factor
TraesCS1D01G387200.1	-1.740	2.2E-139	1.9E-135	-1.724	6.4E-137	1.6E-132	Syntaxin, putative
TraesCS1D01G388300.3	-1.021	5.1E-35	4.4E-32	-1.020	6.8E-35	1.1E-31	DEAD/DEAH box RNA helicase family protein
TraesCS1D01G389000.1	-1.042	2.8E-40	2.7E-37	-1.097	1.3E-44	2.6E-41	ER membrane protein complex subunit 6
TraesCS1D01G389500.1	-0.522	6.5E-09	2.0E-06	-0.445	7.2E-07	3.9E-04	Cyclin-D1-binding protein 1
TraesCS1D01G389900.1	-0.868	8.0E-23	4.6E-20	-0.738	6.6E-17	6.4E-14	GDSL esterase/lipase
TraesCS1D01G390500.1	-0.459	1.5E-12	6.1E-10	-0.478	1.7E-13	1.4E-10	Asparagine synthetase
TraesCS1D01G390600.1	-0.654	2.8E-13	1.2E-10	-0.686	1.8E-14	1.5E-11	Serine/threonine protein phosphatase 2A regulatory subunit B
TraesCS1D01G391000.1	-1.424	2.3E-77	5.6E-74	-1.376	3.7E-72	1.7E-68	Photosynthetic NDH subcomplex B 3
TraesCS1D01G391600.1	-0.416	3.1E-06	7.5E-04	-0.372	2.9E-05	1.1E-02	60S ribosomal protein L35a-like protein
TraesCS1D01G392300.1	-0.780	6.7E-31	5.1E-28	-0.784	3.9E-31	6.0E-28	2-oxoglutarate (2OG) and Fe(II)-dependent oxygenase
TraesCS1D01G393000.2	-0.589	1.9E-16	8.9E-14	-0.592	1.3E-16	1.2E-13	Dynamin-related protein
TraesCS1D01G393500.1	-0.828	1.9E-22	1.1E-19	-0.840	5.0E-23	5.8E-20	O-acyltransferase WSD1
TraesCS1D01G393600.1	-0.778	3.9E-21	2.1E-18	-0.796	4.5E-22	5.0E-19	Cytochrome b561 and DOMON domain-containing protein
TraesCS1D01G394600.1	-0.608	1.4E-11	5.1E-09	-0.507	1.8E-08	1.1E-05	Sodium Bile acid symporter family
TraesCS1D01G394700.1	-0.756	4.6E-18	2.3E-15	-0.736	3.8E-17	3.7E-14	60S ribosomal protein L37a
TraesCS1D01G395700.1	-0.666	9.1E-14	3.8E-11	-0.720	8.0E-16	7.2E-13	ARM repeat superfamily protein
TraesCS1D01G395900.1	-0.898	2.7E-26	1.7E-23	-0.949	3.0E-29	4.2E-26	Ref_Wollemi_Transcript_14910_3291 transcribed RNA sequence
TraesCS1D01G396000.2	-1.183	4.5E-55	6.3E-52	-1.153	2.8E-52	6.3E-49	Ubiquitin-conjugating enzyme E2
TraesCS1D01G396300.1	-0.915	1.3E-47	1.4E-44	-0.783	4.5E-35	7.4E-32	Ubiquitin-conjugating enzyme E2
TraesCS1D01G396400.1	-0.434	1.3E-06	3.4E-04	-0.358	6.7E-05	2.5E-02	50S ribosomal protein L21
TraesCS1D01G396800.1	-1.288	1.3E-60	2.1E-57	-1.231	1.9E-55	5.1E-52	Oxidoreductase
TraesCS1D01G397100.1	-0.540	1.7E-09	5.5E-07	-0.457	3.5E-07	1.9E-04	Ribosomal protein
TraesCS1D01G397300.1	-0.492	4.2E-08	1.2E-05	-0.417	3.2E-06	1.6E-03	Translationally-controlled tumor protein-like protein
TraesCS1D01G397600.1	-0.763	9.0E-19	4.7E-16	-0.720	7.2E-17	6.8E-14	Ninja-family protein
TraesCS1D01G398200.3	-0.731	1.0E-16	4.9E-14	-0.781	7.2E-19	7.4E-16	Disease resistance protein (NBS-LRR class) family
TraesCS1D01G398400.1	-1.193	3.7E-55	5.2E-52	-1.235	4.5E-59	1.3E-55	Auxin-responsive protein
TraesCS1D01G398600.1	-1.309	7.5E-63	1.3E-59	-1.292	3.4E-61	1.1E-57	UV-B-induced protein, chloroplastic
TraesCS1D01G398700.1	-1.151	3.4E-45	3.8E-42	-1.104	1.0E-41	2.1E-38	Protein PLANT CADMIUM RESISTANCE 2
TraesCS1D01G399000.2	-1.077	7.0E-40	6.7E-37	-1.077	7.8E-40	1.5E-36	Glycosyltransferases
TraesCS1D01G399800.2	-0.924	4.6E-26	2.9E-23	-0.948	2.4E-27	3.0E-24	Golgin candidate 5
TraesCS1D01G399900.1	-0.480	8.5E-08	2.5E-05	-0.387	1.5E-05	6.2E-03	Leucine-rich repeat receptor-like protein kinase family protein
TraesCS1D01G400100.1	-2.250	4.1E-275	4.0E-270	-2.326	7.9E-294	8.1E-289	Photosystem I reaction center subunit VI
TraesCS1D01G400500.1	-1.124	9.3E-48	1.1E-44	-1.237	1.0E-57	2.9E-54	Trihelix transcription factor
TraesCS1D01G400600.1	-1.396	3.2E-74	7.3E-71	-1.462	3.1E-81	1.6E-77	Ubiquitin-conjugating enzyme E2
TraesCS1D01G402600.1	-0.613	1.5E-12	6.1E-10	-0.569	5.5E-11	4.0E-08	DNA repair protein RecN
TraesCS1D01G402800.1	-0.350	7.6E-05	1.5E-02	-0.344	9.8E-05	3.4E-02	F-box family protein
TraesCS1D01G403300.1	-0.516	1.0E-08	3.2E-06	-0.611	1.1E-11	8.7E-09	Cytochrome P450 family protein, expressed
TraesCS1D01G403400.1	-1.224	2.0E-121	1.1E-117	-1.275	1.4E-131	2.9E-127	Protein DEHYDRATION-INDUCED 19

TraesCS1D01G403500.1	-0.761	2.5E-17	1.2E-14	-0.648	5.5E-13	4.4E-10	Chaperone protein dnaJ
TraesCS1D01G476000LC.1	-0.569	2.3E-10	8.2E-08	-0.419	3.3E-06	1.6E-03	Heat shock transcription factor
TraesCS1D01G483300LC.1	-0.366	2.9E-05	6.0E-03	-0.386	1.0E-05	4.4E-03	ARM repeat superfamily protein
TraesCS1D01G491300LC.1	-0.630	2.0E-12	7.9E-10	-0.522	5.9E-09	3.7E-06	Cyclic pyranopterin monophosphate synthase
TraesCS1D01G505500LC.1	-0.423	2.6E-06	6.5E-04	-0.455	4.4E-07	2.4E-04	Protein translocase subunit SecA
TraesCS1D01G507900LC.1	-0.854	5.3E-92	1.8E-88	-0.841	4.6E-89	2.9E-85	ATP synthase subunit beta
TraesCS1D01G525200LC.1	-0.345	6.3E-05	1.2E-02	-0.466	5.7E-08	3.3E-05	DNA repair and meiosis protein (Mre11)
TraesCS2A01G158900.6	0.392	7.1E-07	1.9E-04	0.390	7.7E-07	4.1E-04	Myb-related protein-like
TraesCS2A01G233800.2	0.707	1.2E-15	5.3E-13	0.704	1.5E-15	1.3E-12	Nucleobase ascorbate transporter
TraesCS2B01G285200LC.1	0.518	1.8E-10	6.3E-08	0.555	1.0E-11	8.0E-09	Phosphatidylserine decarboxylase proenzyme
TraesCS2B01G612800LC.1	0.403	1.8E-06	4.5E-04	0.341	5.6E-05	2.1E-02	RAD3-like DNA-binding helicase protein
TraesCS2D01G266100.1	0.282	7.3E-05	1.4E-02	0.281	7.6E-05	2.7E-02	Tobamovirus multiplication 1
TraesCS2D01G356700.2	0.307	2.8E-05	5.8E-03	0.305	2.9E-05	1.1E-02	Dol-P-Glc:Glc(2)Man(9)GlcNAc(2)-PP-Dol alpha-1,2-glucosyltransferase
TraesCS3A01G547400LC.1	0.429	1.0E-07	2.9E-05	0.427	1.1E-07	6.3E-05	50S ribosomal protein L24
TraesCS3B01G449100LC.1	0.837	9.9E-21	5.4E-18	0.843	5.3E-21	5.8E-18	E3 ubiquitin-protein ligase
TraesCS3B01G449200LC.1	0.378	3.0E-06	7.4E-04	0.351	1.4E-05	6.0E-03	inositol transporter 4
TraesCS3B01G635500LC.1	-0.364	4.1E-05	8.3E-03	-0.407	4.4E-06	2.1E-03	Mediator of RNA polymerase II transcription subunit 24
TraesCS3B01G662500LC.1	0.491	4.0E-10	1.4E-07	0.519	5.3E-11	3.8E-08	aspartate kinase-homoserine dehydrogenase ii
TraesCS3D01G099600.3	0.356	3.5E-06	8.3E-04	0.355	3.7E-06	1.8E-03	Breast cancer type 2 susceptibility protein-like protein
TraesCS3D01G230800.4	0.384	8.0E-07	2.1E-04	0.383	8.4E-07	4.5E-04	CENP-C
TraesCS4B01G059500.1	0.335	5.7E-06	1.3E-03	0.334	5.8E-06	2.7E-03	ethylene-dependent gravitropism-deficient and yellow-green-like 2
TraesCS5A01G553900LC.1	-0.450	4.3E-07	1.2E-04	-0.384	1.6E-05	6.4E-03	spatacsin carboxy-terminus protein
TraesCS5B01G304600.1	0.885	6.5E-23	3.8E-20	0.880	1.1E-22	1.2E-19	F-box protein
TraesCS5B01G442400LC.1	0.263	9.6E-08	2.8E-05	0.232	2.4E-06	1.2E-03	Bis(5'-nucleosyl)-tetraphosphatase, symmetrical
TraesCS6A01G206600.1	-0.479	2.0E-23	1.2E-20	-0.409	2.2E-17	2.1E-14	Photosynthetic NDH subcomplex L 2
TraesCS6B01G409300LC.1	0.332	3.7E-05	7.6E-03	0.355	9.1E-06	3.9E-03	Transducin family protein / WD-40 repeat family protein
TraesCS7A01G170000.1	-0.432	1.4E-06	3.6E-04	-0.401	7.7E-06	3.5E-03	Myb/SANT-like DNA-binding domain protein
TraesCS7A01G378600LC.1	-0.571	1.6E-11	5.7E-09	-0.523	7.6E-10	5.1E-07	Bifunctional protein FolD
TraesCS7B01G350200.1	0.378	2.5E-07	7.1E-05	0.328	7.0E-06	3.2E-03	Calcium-binding family protein

Supplementary Table 11. Homeologous regions on chromosome group 1 experiencing segmental coregulation.

	Start		End			
**Chr**	**Gene**	**Position**	**Gene**	**Position**	**Size**	**Modification**
1A	TraesCS1A01G350500	535.3 Mb	TraesCS1A01G395300	561.0 Mb	25.7 Mb	Suppression
1B	TraesCS1B01G364800	595.2 Mb	TraesCS1B01G423600	646.2 Mb	51.0 Mb	None
1D	TraesCS1D01G353300	439.0 Mb	TraesCS1D01G403500	467.9 Mb	28.9 Mb	Induction

Supplementary Table 12 | Differentially expressed genes between *Pgt*-inoculated and mock-inoculated in NS1 at 24 hpi.

	CTH Mock v *Pgt*			NS1 Mock vs *Pgt*			NS2 M v Pgt 24			
**Gene**	**Fold change (log2)**	***p*-value**	***q*-value**	**Fold change (log2)**	***p*-value**	***q*-value**	**Fold change (log2)**	***p*-value**	***q*-value**	**Description**
TraesCS1A01G203600.1	-2.78	1.7E-15	2.2E-11	-1.82	2.8E-07	1.4E-03	-0.99	4.4E-03	5.2E-02	Chitinase
TraesCS1B01G245800.1	0.92	4.2E-01	6.6E-01	6.59	1.4E-05	2.6E-02	-1.93	7.6E-02	NA	NRR repressor homolog 1
TraesCS1B01G308900.1	-0.86	3.2E-01	5.6E-01	-3.74	2.5E-05	3.5E-02	-0.89	3.0E-01	5.9E-01	Aminotransferase like protein
TraesCS1B01G368700LC.1	-0.38	1.3E-01	3.3E-01	-1.05	4.2E-05	4.8E-02	-0.68	6.1E-03	6.5E-02	Mitogen-activated protein kinase kinase kinase 1
TraesCS1B01G425000.1	-0.85	7.2E-01	8.7E-01	17.27	9.8E-11	1.0E-06	19.42	1.3E-13	NA	Fasciclin-like arabinogalactan protein
TraesCS1D01G292100.1	-2.24	4.3E-03	3.3E-02	-3.75	2.8E-06	8.9E-03	-1.07	1.7E-01	4.4E-01	Aminotransferase like protein
TraesCS2A01G130600.2	21.07	1.8E-06	1.0E-04	-19.90	6.6E-06	1.6E-02	0.28	9.5E-01	NA	Glutamate synthase, putative
TraesCS2A01G316800LC.1	40.20	2.0E-08	3.1E-06	-36.39	3.8E-07	1.9E-03	0.16	9.8E-01	9.9E-01	phosphatidyl inositol monophosphate 5 kinase
TraesCS2A01G354300LC.1	0.60	2.3E-02	1.0E-01	1.30	6.6E-07	3.0E-03	1.68	4.9E-10	3.9E-07	Disease resistance protein (TIR-NBS-LRR class) family
TraesCS2A01G419900.1	-1.21	1.1E-01	2.9E-01	6.70	1.7E-06	6.0E-03	-0.43	5.7E-01	NA	Argonaute family protein
TraesCS2B01G314000.1	19.32	1.4E-04	2.7E-03	-22.62	7.4E-06	1.7E-02	0.02	1.0E+00	NA	Digalactosyldiacylglycerol synthase 1, chloroplastic
TraesCS2D01G062500LC.1	2.03	1.7E-05	5.9E-04	-1.75	1.1E-05	2.2E-02	0.85	9.7E-02	3.2E-01	DNAse I-like superfamily protein
TraesCS2D01G481900.2	-0.50	5.7E-01	NA	5.99	2.4E-05	3.5E-02	1.31	1.2E-01	NA	Anthocyanidin reductase
TraesCS3A01G120200.1	-1.63	5.9E-01	7.9E-01	-21.15	5.1E-11	6.3E-07	-1.11	7.1E-01	NA	Valine--tRNA ligase
TraesCS3A01G229100.1	-0.32	2.0E-01	4.3E-01	-1.11	6.4E-06	1.6E-02	-0.04	8.8E-01	9.5E-01	Kinesin-like protein
TraesCS3B01G113500.1	0.67	9.1E-01	9.6E-01	-48.96	4.0E-16	8.4E-12	46.56	1.0E-14	NA	Nuclease
TraesCS3B01G256800.1	-0.06	8.6E-01	9.4E-01	-1.67	3.8E-06	1.1E-02	-0.04	9.2E-01	9.7E-01	Siroheme synthase
TraesCS3B01G324500.2	-0.66	6.8E-02	2.2E-01	-2.02	3.1E-06	9.1E-03	-0.32	3.1E-01	5.9E-01	Cellulose synthase
TraesCS3B01G578200.1	-0.86	2.6E-01	5.1E-01	6.26	4.9E-06	1.3E-02	1.38	7.3E-02	NA	Abscisic stress ripening protein
TraesCS3D01G193000.1	-0.73	8.3E-02	2.5E-01	-2.43	7.1E-06	1.6E-02	0.24	5.2E-01	NA	Transmembrane protein, putative
TraesCS3D01G248700.1	-0.01	1.0E+00	1.0E+00	7.88	3.0E-05	4.2E-02	1.23	4.1E-01	NA	Myosin
TraesCS3D01G370500.1	0.24	7.0E-01	8.6E-01	6.25	2.1E-06	6.9E-03	0.28	6.6E-01	NA	Histone H3
TraesCS3D01G494600.2	-0.61	1.0E-02	6.1E-02	-1.03	2.7E-05	3.7E-02	-0.79	3.1E-04	8.6E-03	Response regulator
TraesCS4A01G259400.1	-0.55	3.3E-01	5.8E-01	2.43	3.9E-05	4.8E-02	-0.14	8.0E-01	9.2E-01	Anthocyanin 5-aromatic acyltransferase
TraesCS4B01G028000.1	0.30	4.5E-01	6.9E-01	-1.73	1.8E-05	3.0E-02	0.71	9.7E-02	3.2E-01	Integral membrane HPP family protein
TraesCS4D01G015200LC.1	1.61	1.6E-09	4.2E-07	-1.08	2.1E-05	3.2E-02	0.72	1.3E-02	1.0E-01	Serine/threonine-protein kinase atg1
TraesCS4D01G135000.6	2.02	1.6E-01	3.8E-01	11.49	6.7E-10	6.0E-06	-0.89	5.4E-01	7.8E-01	Ribulose bisphosphate carboxylase/oxygenase activase
TraesCS4D01G208200.2	0.32	7.9E-01	9.0E-01	-6.79	3.9E-05	4.8E-02	6.59	6.6E-05	NA	S-acyltransferase
TraesCS4D01G228600.2	-0.69	4.6E-01	6.9E-01	-5.87	3.9E-05	4.8E-02	-1.35	1.6E-01	NA	Ras-related protein Rab-18
TraesCS4D01G284300.1	-1.14	2.9E-03	2.5E-02	-2.12	2.3E-05	3.5E-02	-0.29	3.5E-01	NA	F-box and associated interaction domains-containing protein
TraesCS5A01G060600.1	0.31	6.7E-02	2.1E-01	0.70	2.5E-05	3.5E-02	0.43	9.9E-03	8.7E-02	Calcineurin B-like protein
TraesCS5A01G146500.1	2.22	2.1E-01	4.4E-01	6.14	2.0E-05	3.2E-02	1.90	2.4E-01	5.2E-01	WRKY transcription factor
TraesCS5A01G226400.1	-1.99	1.2E-05	4.4E-04	-1.99	1.6E-05	2.8E-02	-0.75	9.7E-02	3.2E-01	2-oxoglutarate (2OG) and Fe(II)-dependent oxygenase
TraesCS5A01G355100.1	-0.36	1.1E-01	2.9E-01	-1.06	1.5E-05	2.7E-02	-0.39	7.9E-02	2.9E-01	ecotropic viral integration site protein
TraesCS5A01G455800.2	0.50	2.7E-03	2.4E-02	0.71	1.5E-05	2.7E-02	0.47	8.0E-03	7.7E-02	Pheophorbide a oxygenase, chloroplastic
TraesCS5B01G224100.1	-0.19	8.3E-01	9.2E-01	3.44	3.4E-05	4.5E-02	-0.89	2.7E-01	NA	WRKY transcription factor

TraesCS5B01G400000.2	-1.43	6.9E-01	8.5E-01	-22.55	1.7E-09	1.2E-05	0.07	9.8E-01	NA	Alpha-1,4 glucan phosphorylase
TraesCS5D01G005300.2	-1.49	3.0E-01	5.5E-01	-9.07	1.0E-06	4.1E-03	-0.18	9.0E-01	9.6E-01	Disease resistance protein (NBS-LRR class) family
TraesCS5D01G020400.3	0.99	4.3E-01	6.7E-01	7.39	1.6E-05	2.7E-02	0.87	4.9E-01	NA	WD-repeat protein, putative
TraesCS5D01G069600.1	0.48	1.7E-04	3.2E-03	0.52	4.1E-05	4.8E-02	0.54	4.2E-05	2.0E-03	Translocase subunit seca
TraesCS5D01G132900.2	-0.62	5.0E-01	7.3E-01	6.74	5.7E-06	1.5E-02	-0.35	7.1E-01	NA	Transcription factor-related family protein
TraesCS5D01G434600.1	-1.11	2.0E-02	9.6E-02	-3.22	1.2E-05	2.4E-02	0.22	6.2E-01	8.2E-01	Fasciclin-like arabinogalactan protein
TraesCS6A01G314100.2	-19.69	5.2E-10	1.8E-07	-18.66	5.0E-09	3.1E-05	0.03	9.9E-01	NA	Peptidylprolyl isomerase
TraesCS6A01G394800.2	-1.25	6.8E-01	8.5E-01	-18.39	1.7E-08	9.5E-05	0.03	9.9E-01	NA	OTU domain-containing protein
TraesCS6B01G309400.2	-2.95	7.1E-03	4.7E-02	6.14	3.9E-05	4.8E-02	5.87	8.9E-05	NA	Fatty acid desaturase
TraesCS6B01G324700.4	-34.15	1.9E-06	1.1E-04	34.36	1.6E-06	6.0E-03	2.98	6.7E-01	NA	Mediator of RNA polymerase II transcription subunit 23
TraesCS6D01G157000.1	-1.81	6.0E-02	2.0E-01	6.61	8.4E-06	1.8E-02	-0.33	7.1E-01	NA	Brefeldin A-inhibited guanine nucleotide-exchange protein
TraesCS6D01G359300.1	-2.71	8.2E-07	5.7E-05	-2.74	7.6E-07	3.2E-03	-0.72	1.9E-01	4.6E-01	2-oxoglutarate (2OG) and Fe(II)-dependent oxygenase
TraesCS7A01G350300.1	3.20	3.2E-01	5.7E-01	-20.58	9.2E-10	7.2E-06	-1.57	6.2E-01	NA	Cyclic nucleotide-gated ion channel, putative
TraesCS7A01G368300.2	-0.32	8.6E-01	9.4E-01	-19.79	4.2E-21	1.3E-16	1.81	3.0E-01	NA	Dehydrogenase/reductase
TraesCS7B01G225500.2	1.52	1.6E-01	3.8E-01	-6.56	3.2E-05	4.3E-02	-0.82	4.4E-01	7.1E-01	Transposon protein, putative, mutator sub-class
TraesCS7B01G296500.4	39.75	1.1E-41	6.0E-37	38.58	2.1E-39	1.3E-34	-33.64	2.2E-27	NA	Processing peptidase
TraesCS7B01G749100LC.1	-0.31	5.5E-01	7.6E-01	-5.70	1.0E-05	2.1E-02	-2.08	1.7E-04	5.5E-03	Aspartyl protease family protein 2
TraesCS7D01G227400.1	0.01	1.0E+00	1.0E+00	-20.90	3.8E-13	5.9E-09	-0.54	8.4E-01	NA	UPF0061 protein
TraesCSU01G029700.1	-1.20	7.8E-02	2.4E-01	-3.18	4.1E-05	4.8E-02	0.03	9.6E-01	NA	Trihelix transcription factor GT-2

Supplementary Table 13 | *De novo* transcriptome assemblies of several grass species.

Species	Assembly	Reference
Achnatherum splendens	https://doi.org/10.6084/m9.figshare.5601565	[82]
Agropyron desertorum	https://doi.org/10.6084/m9.figshare.5601412	NCBI PRJNA307648
Agrostis stolonifera	https://doi.org/10.6084/m9.figshare.5601418	NCBI PRJNA304034
Bromus inermis	https://doi.org/10.6084/m9.figshare.5601427	NCBI PRJNA307648
Dactylis glomerata	https://doi.org/10.6084/m9.figshare.5601436	NCBI PRJEB16763
Festuca pratensis	https://doi.org/10.6084/m9.figshare.5601499	NCBI PRJNA308063
Holcus lanatus	https://doi.org/10.6084/m9.figshare.5601502	[93]
Melica nutans	https://doi.org/10.6084/m9.figshare.5601505	[81]

Supplementary Table 14 | Pairwise comparison of nucleotide and protein sequences of *Med15* homologs.

Med15	*OsMed15*	*BdMed15*	*BiMed15*	*HvMed15a*	*TaMed15a.A*	*TaMed15a.B*	*TaMed15a.D*	*HvMed15b*	*TaMed15b.D*	*TaMed15b.A*	*TaMed15b.B*
OsMed15	-	78.8	78.1	79.5	78.6	79.4	79.3	70.6	77.1	76.7	76.9
BdMed15	81.1	-	82.1	83.3	82.6	83.4	83.1	76.0	80.5	80.0	80.0
BiMed15	81.0	84.6	-	90.6	90.5	91.1	91.0	78.2	86.1	85.7	85.3
HvMed15a	80.8	85.2	93.0	-	94.7	95.3	95.0	79.4	87.1	86.7	86.4
TaMed15a.A	80.7	85.1	92.9	95.7	-	97.7	97.5	79.1	87.4	86.8	86.5
TaMed15a.B	81.3	85.8	93.6	96.5	98.3	-	98.1	79.6	87.9	87.3	87.0
TaMed15a.D	81.2	85.6	93.5	96.3	98.1	98.9	-	79.5	87.7	87.1	86.8
HvMed15b	74.0	79.1	82.2	82.6	82.8	83.4	83.3	-	79.7	79.4	79.6
TaMed15b.D	79.0	82.9	88.9	89.7	89.8	90.5	90.3	83.6	-	97.8	97.4
TaMed15b.A	78.8	82.7	88.9	89.5	89.7	90.3	90.1	83.4	97.7	-	97.2
TaMed15b.B	78.9	82.3	88.4	89.3	89.4	90.1	89.9	83.2	97.5	97.4	-

Numbers above the dashes designate nucleotide identity of the open reading frame, whereas numbers below the dashes designate protein identity.
