# Supplementary material for: Stem rust resistance in wheat is suppressed by a subunit of the mediator complex
Source: Nat Commun. 2020 Feb 28;11:1123. doi: 10.1038/s41467-020-14937-2 (PMC7048732; doi:10.1038/s41467-020-14937-2)
Supplement: Supplementary file 3 — Reporting Summary [file 41467_2020_14937_MOESM3_ESM.pdf]

nature research

Corresponding author(s): Colin Hiebert, Matthew Moscou, Evans Lagudah, Wolfgang Spielmeyer

Last updated by author(s): Feb 4, 2020

Reporting Summary

Nature Research wishes to improve the reproducibility of the work that we publish. This form provides structure for consistency and transparency in reporting. For further information on Nature Research policies, see Authors & Referees and the Editorial Policy Checklist .

Statistics

For all statistical analyses, confirm that the following items are present in the figure legend, table legend, main text, or Methods section.

n/a	Confirmed	
☐	☒	The exact sample size (n) for each experimental group/condition, given as a discrete number and unit of measurement
☐	☒	A statement on whether measurements were taken from distinct samples or whether the same sample was measured repeatedly
☐	☒	The statistical test(s) used AND whether they are one- or two-sided<br>*Only common tests should be described solely by name; describe more complex techniques in the Methods section.*
☒	☐	A description of all covariates tested
☐	☒	A description of any assumptions or corrections, such as tests of normality and adjustment for multiple comparisons
☐	☒	A full description of the statistical parameters including central tendency (e.g. means) or other basic estimates (e.g. regression coefficient) AND variation (e.g. standard deviation) or associated estimates of uncertainty (e.g. confidence intervals)
☐	☒	For null hypothesis testing, the test statistic (e.g. F, t, r) with confidence intervals, effect sizes, degrees of freedom and P value noted<br>*Give P values as exact values whenever suitable.*
☒	☐	For Bayesian analysis, information on the choice of priors and Markov chain Monte Carlo settings
☐	☒	For hierarchical and complex designs, identification of the appropriate level for tests and full reporting of outcomes
☒	☐	Estimates of effect sizes (e.g. Cohen's d, Pearson's r), indicating how they were calculated

Our web collection on statistics for biologists contains articles on many of the points above.

Software and code

Policy information about availability of computer code

Data collection	No software was used for data collection.
Data analysis	Statistical analyses were performed using R version 3.5.1. Trimmomatic version v0.36 was used for trimming Illumina reads. De novo assembly of Canthatch, NS1, and NS2 was performed using Edena v3.131028. RepeatMasker version open-4.0.5 using repeat library version 20140131 was used for repeat masking. Alignments and analysis of aligned reads to the genomic sequence was performed using bwa version 0.7.10-r789 and samtools version 1.9. Nucleotide polymorphisms were performed using VarScan 2.3.8. Tophat version 2.0.9 was used for splice alignment of RNAseq reads to genomic sequence. TransDecoder version 2.0.1 was used for ORF prediction. BLAST version 2.2.26 was used for sequence comparison. Genetic map development was performed using MapDisto version 2.1. Additional genome assemblies were performed with CLC Genomics Workbench (v9), RepeatMasker v4.0.6 using the custom Triticeae, non-redundant repeat library trep-db_nr_Rel-16.fasta (release 16), alignments with bwa v0.7.15, and samtools version v0.1.19. De novo transcriptome assemblies were performed using Trinity r20140717. PRANK (v.140603) and RAxML (v8.2.9) were used for codon-based alignment and phylogenetic tree construction. Molecular evolutionary analyses were performed with PAML (v4.8). RNAseq analysis was performed using Kallisto (v0.43.1), BBmap (v37.77), and DEseq2 (Bioconductor v3.2). Custom scripts and analyses are available through the Github repositories https://github.com/matthewmoscou/Canthatch, https://github.com/matthewmoscou/QKutilities, and https://github.com/TC-Hewitt/MuTrigo.

For manuscripts utilizing custom algorithms or software that are central to the research but not yet described in published literature, software must be made available to editors/reviewers. We strongly encourage code deposition in a community repository (e.g. GitHub). See the Nature Research guidelines for submitting code & software for further information.

Data

Policy information about availability of data

All manuscripts must include a data availability statement. This statement should provide the following information, where applicable:

- Accession codes, unique identifiers, or web links for publicly available datasets
- A list of figures that have associated raw data
- A description of any restrictions on data availability

All high-throughput sequencing data described in this manuscript have been deposited in the NCBI BioProject PRJNA401266 and European Nucleotide Archive (ENA) PRJEB23265. Illumina sequencing data for flow-sorted chromosomes are deposited in sequential NCBI SRR accessions SRR6001708 to SRR6001719 and ENA ERS1988338 to ERS1988347, de novo assemblies of flow-sorted chromosomes for CTH-K (NCBI NTGG00000000, ENA ERS1988348), CTH-W (ENA ERS1988349), NS1 (NCBI NTGH00000000), and NS2 (NCBI NTGI00000000). RNAseq sequencing data are deposited in NCBI SRR accessions SRR6003621 to SRR6003628 and SRR10426855 to SRR10426890.

Field-specific reporting

Please select the one below that is the best fit for your research. If you are not sure, read the appropriate sections before making your selection.

☒ Life sciences ☐ Behavioural & social sciences ☐ Ecological, evolutionary & environmental sciences

For a reference copy of the document with all sections, see nature.com/documents/nr-reporting-summary-flat.pdf

Life sciences study design

All studies must disclose on these points even when the disclosure is negative.

Sample size	Sample sizes are reported. We used genetic populations for linkage mapping and a set of mutants for identifying the gene of interest. For the genetic populations we generated doubled haploid populations for which there are practical limits on population size. Both populations are within the range commonly found for linkage mapping experiments. The number of mutants used in this study were equal or greater than the number of mutants typically reported in the literature for gene isolation experiments.
Data exclusions	No data was excluded.
Replication	Testing mutants and genetic populations with stem rust (fungal disease) was replicated and several plants were tested with each replicate. All RNAseq experiments were performed in triplicate. For growth assays, six individual plants were evaluated in three independent environments.
Randomization	The seedling stem rust tests we conducted were indoors, controlled tests that produced qualitative responses. Experiments of this nature are generally not randomized in published studies. As such, our populations were not randomized for these indoor studies. For the field tests, quantitative data on disease severity was collected and the tests were randomized (randomized complete block design). For RNAseq experiments, samples were randomized and replicated. For growth assays, all plants were randomized within the greenhouse and assays performed over three different environments.
Blinding	Seedlings in the greenhouse and field plots were issued sequential plant/plot numbers. The researcher rating disease symptoms had no knowledge of the previous ratings or any DNA marker genotypes of the plants being assessed. Thus, the rating was blind. RNAseq and growth assays were randomized and blind.

Reporting for specific materials, systems and methods

We require information from authors about some types of materials, experimental systems and methods used in many studies. Here, indicate whether each material, system or method listed is relevant to your study. If you are not sure if a list item applies to your research, read the appropriate section before selecting a response.

Materials & experimental systems

n/a	Involved in the study	
☒	☐	Antibodies
☒	☐	Eukaryotic cell lines
☒	☐	Palaeontology
☒	☐	Animals and other organisms
☒	☐	Human research participants
☒	☐	Clinical data

Methods

n/a	Involved in the study	
☒	☐	ChIP-seq
☐	☒	Flow cytometry
☒	☐	MRI-based neuroimaging

Flow Cytometry

Plots

Confirm that:

- [x] The axis labels state the marker and fluorochrome used (e.g. CD4-FITC).
- [x] The axis scales are clearly visible. Include numbers along axes only for bottom left plot of group (a 'group' is an analysis of identical markers).
- [ ] All plots are contour plots with outliers or pseudocolor plots.
- [ ] A numerical value for number of cells or percentage (with statistics) is provided.

Methodology

Sample preparation	Suspensions of intact mitotic metaphase chromosomes were prepared according to Vrana et al. (2000) from synchronized root tips of young seedlings of the following lines: CTH-K, NS1, NS2, CTH-W, mutants W01, W02, W06, W07, W10, Thatcher, and CTH-DT7DL. Prior to chromosome analysis by flow cytometry, fluorescence in situ hybridization in suspension (FISHIS) was used to label GAA microsatellites by FITC following the protocol of Giorgi et al. (2013). Briefly, chromosomal DNA was denatured by adding 10 M NaOH to the solution to reach pH 12.8 - 13.3. Following incubation at room temperature for 15 min, pH of the solution was changed to 8.5 - 9.1 using 1M Tris-HCl (pH 7.5) and the sample was incubated on ice for 1 min. Then, (GAA)7-FITC probe was added to the suspension to the final concentration 4.6 ng/uL and the sample was incubated in darkness at room temperature for 1 hour. After FISHIS, chromosomal DNA was stained by fluorochrome DAPI (4',6-diamidino-2-phenylindole) at 2 uL/mL final concentration.
Instrument	FACSAria II SORP, BD Biosciences, San Jose, CA, USA; Firmware ver. 1.6 (BD FACSAria II)
Software	FACS Diva, ver. 6.1.3
Cell population abundance	*Describe the abundance of the relevant cell populations within post-sort fractions, providing details on the purity of the samples and how it was determined.*
Gating strategy	*Describe the gating strategy used for all relevant experiments, specifying the preliminary FSC/SSC gates of the starting cell population, indicating where boundaries between "positive" and "negative" staining cell populations are defined.*

- [ ] Tick this box to confirm that a figure exemplifying the gating strategy is provided in the Supplementary Information.
